# Unveiling the Bioactive Efficacy of *Cupressus sempervirens* ‘Stricta’ Essential Oil: Composition, In Vitro Activities, and In Silico Analyses

**DOI:** 10.3390/ph17081019

**Published:** 2024-08-02

**Authors:** Eman Fikry, Raha Orfali, Nora Tawfeek, Shagufta Perveen, Safina Ghafar, Maher M. El-Domiaty, Azza M. El-Shafae

**Affiliations:** 1 Department of Pharmacognosy, Faculty of Pharmacy, Zagazig University, Zagazig 44519, Egypt; efhassan@zu.edu.eg (E.F.); mmeldomyaty@pharmacy.zu.edu.eg (M.M.E.-D.); amelshafaey@pharmacy.zu.edu.eg (A.M.E.-S.); 2 Department of Pharmacognosy, Collage of Pharmacy, King Saud University, P.O. Box 2457, Riyadh 11451, Saudi Arabia; 3 Department of Bacteriology, University of Wisconsin-Madison, Madison, WI 53706, USA

**Keywords:** *Cupressus sempervirens* ‘Stricta’, MTT assay, antidiabetic, antioxidant, antimicrobial, network pharmacology, gene expression, molecular docking

## Abstract

Prior studies have extensively investigated the essential oil derived from the Mediterranean cypress, *Cupressus sempervirens*. However, the ‘Stricta’ variety, known for its ornamental value, has received less attention in terms of its oil composition and potential health benefits. The objective of this research was to comprehensively analyze the chemical components and medicinal properties of the essential oil extracted from *C. sempervirens* ‘Stricta’ (CSSLEO) grown in Egypt. Utilizing gas chromatography–mass spectrometry (GC–MS), the investigation identified 22 compounds within CSSLEO, with *α*-pinene and *δ*-3-carene being predominant, accounting for 96.01% of the oil. In vitro assays evaluated CSSLEO’s cytotoxic effects on cancer cell lines, revealing notable anticancer potential. Additionally, the oil displayed antidiabetic properties by impeding crucial enzymes involved in glucose metabolism. Complementary in silico network pharmacology and molecular docking studies provided insights into the possible interactions between CSSLEO’s key compounds and essential proteins and pathways in cancer treatment. The results underscored CSSLEO’s intricate composition and its promising applications in cancer prevention and diabetes management. The conclusions drawn from this research underscore the need for further investigation to validate CSSLEO’s clinical effectiveness and to gain a deeper understanding of its therapeutic mechanisms, with a view to harnessing its potential in oncology and endocrinology.

## 1. Introduction

Essential oils (EOs) have been utilized for centuries due to their medicinal properties and have played a crucial role in the culinary world by adding unique aromas and flavors, as well as serving as natural preservatives [1]. These oils are intricate combinations of various chemical constituents, each possessing distinct functional groups and molecular structures [2]. As secondary metabolites found in plants, EOs are primarily responsible for the biological activities exhibited by aromatic plants. Numerous in vitro and in vivo studies have confirmed the diverse functions of EOs and their individual components, showcasing antioxidant, antimicrobial, anti-inflammatory, antidiabetic, antiviral, cytotoxic, antihypertensive, antihyperlipidemic, and antispasmodic properties through different mechanisms [3,4,5].

Gymnosperms are known for their abundance of essential oil (EO) components, such as terpenoids, phenolic compounds, alkanes, and alkenes. These plants have been acknowledged for their numerous health benefits, including their anticancer, antimicrobial, antifungal, and antioxidant activities [6,7,8]. Among the gymnosperms, conifers, particularly those in the Cupressaceae family, are noteworthy as significant sources of EOs [9].

The Cupressaceae family consists of around 30 genera and 134 species, which are distributed globally. These evergreen, resinous trees and shrubs can be either monoecious or dioecious [10]. EOs play a crucial role as chemotaxonomic markers within this family [11,12,13,14], with the genus *Cupressus* being characterized by the predominance of monoterpenes, sesquiterpenes, and diterpenes in their EO profiles [13].

Cypress, known by its classical Latin name *Cupressus*, is native to Asia Minor and Northern Persia and extends westwards into Greece and Crete. The original species, *Cupressus sempervirens*, commonly known as the Mediterranean cypress, has a wide, uneven, spreading crown. This species has a rich history of traditional uses and has been noted for its medicinal properties including cytotoxic, antidiabetic, antioxidant, and antimicrobial activities [13,15,16,17,18].

However, through horticultural development, a strain with more upright branching was selectively bred, resulting in the columnar form known as variety ‘Stricta’ [19]. *Cupressus sempervirens* ‘Stricta’ is a variety found in the central Mediterranean countries, especially Italy, where it has been used as an ornamental plant in landscapes for centuries. This tree can grow up to 25 m tall, characterized by a single upright trunk and numerous closely-spaced vertical branches covered in dense, dark-green foliage [20,21]. Despite being widely cultivated, there is a lack of research on the chemical composition and biological properties of its essential oil, making it an area that requires further exploration and study.

Essential oils (EOs) derived from the *Cupressus* genus have demonstrated antimicrobial, anticancer, and antioxidant properties. For instance, EOs from *C. arizonica* have shown modest antioxidant potential in DPPH assays, limited antioxidant effects in deoxyribose degradation assays, and moderate effects in non-enzymatic lipid peroxidation assays [13]. The Tunisian *C. sempervirens* EO has exhibited significant antioxidant activities in ABTS and DPPH assays, surpassing the effects of 2, 6-di-tert-butyl-4-hydroxy-boxylic acid (BHT) [22]. Additionally, *Cupressus sempervirens* var. Pyramidalis has demonstrated antiproliferative activity against C32 amelanotic melanoma cells [23]. Given these findings, it was imperative to elucidate the chemical composition of EOs produced by *Cupressus sempervirens* ‘Stricta’ (CSSLEO) and to investigate its potential biological activities in vitro, including cytotoxic, antioxidant, antidiabetic, and antimicrobial activities. Furthermore, this research aimed to leverage target network analysis and in silico validation to discover novel medicinal applications for EOs, thereby contributing to the global effort to address various health challenges through natural remedies.

## 2. Results

### 2.1. Chemical Composition of CSSLEO

**Figure 1:**
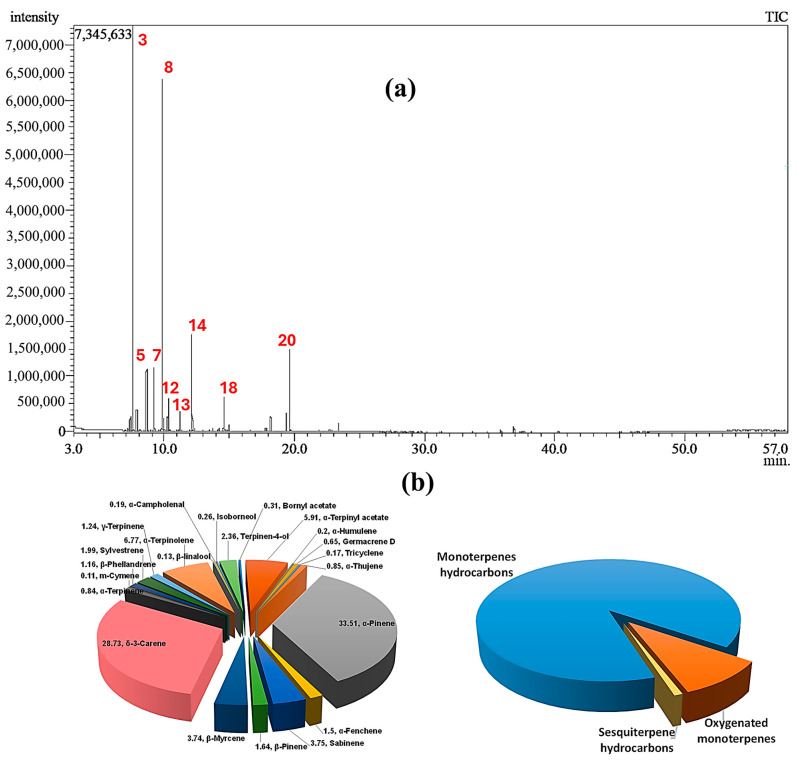
The GC–MS chromatogram (**a**) and the pie charts (**b**) illustrate the relative chemical compositions of *Cupressus sempervirens* ‘Stricta’ leaf essential oil. The red numbers correspond to Table 1.

A transparent, light yellow essential oil (EO) was obtained through the hydrodistillation of fresh leaves from *Cupressus sempervirens* ‘Stricta’. The average yield of the EO was found to be 0.65 ± 0.03% *v*/*w*, based on three separate extractions. Analysis using GC–MS identified a total of 22 components, which accounted for 96.01% of the overall composition. The chemical names and corresponding area percentages of these components can be found in Table 1. Figure 1 displays the GC–MS spectrum and pie chart representing the composition of the EO. The chemical structures of the identified constituents are illustrated in Figure S1.

### 2.2. In Vitro Assessments

#### 2.2.1. Cytotoxic Potential and Selectivity of CSSLEO

The cytotoxic impact of CSSLEO on different human carcinoma cell lines including breast (MCF-7), colon (HCT-116), hepatocellular (HepG-2), and lung (A-549) carcinoma cell lines, as well as the normal African green monkey kidney cell line (Vero), was evaluated through the MTT assay, as shown in Table 2 and Figure S2. CSSLEO exhibited notable cytotoxicity towards all the cancer cell lines tested, with HepG-2 showing the highest sensitivity (IC_50_ = 4.44 μg/mL) compared to HCT-116, A-549, and MCF-7 (IC_50_ = 6.29, 6.71, and 7.20 μg/mL, respectively). Notably, CSSLEO demonstrated selectivity against the carcinoma cell lines (SI > 3) when compared to Vero, as outlined in Table 2.

#### 2.2.2. Antidiabetic Activity

##### α-Glucosidase Inhibition Activity

The inhibitory effect of CSSLEO on *α*-glucosidase was illustrated in Table S1, where it was observed that the level of inhibition increased with higher concentrations of CSSLEO. The calculated IC_50_ value of CSSLEO (100.06 ± 2.18 μg/mL) indicated a substantial inhibitory activity, in comparison to the IC_50_ values of the acarbose standard (2.12 ± 0.54 μg/mL).

##### α-Amylase Inhibition Activity

In Table S2, the potential of CSSLEO to inhibit α-amylase was demonstrated. The extent of enzyme inhibition was directly influenced by the concentration of the tested oil. The IC_50_ value revealed that CSSLEO has half the inhibition potential of acarbose. More specifically, the IC_50_ value for CSSLEO is 26.36 ± 0.91 μg/mL, while acarbose has an IC_50_ value of 12.31 ± 0.87 μg/mL.

#### 2.2.3. Antioxidant Activity

The antioxidant capacity and reducing power of CSSLEO were evaluated using DPPH, ABTS, and FRAP assays, and the results are detailed in Table 3. Unlike ascorbic acid, CSSLEO exhibited lower scavenging abilities against DPPH and ABTS radicals, along with decreased capacity to reduce ferric ions, suggesting a comparatively weaker antioxidant performance.

#### 2.2.4. Antimicrobial Activity

The effectiveness of antimicrobial activity against different fungal and bacterial strains was assessed through the well diffusion method, where the diameter of the inhibition zone (IZ) was measured, and the MIC was determined, as outlined in Table 4.

### 2.3. Network Pharmacology-Based Analysis

#### 2.3.1. Drug-Likeness Screening of the Identified Compounds and Acquiring Compound and Disease-Related Targets

Screening pharmacokinetic parameters plays a crucial role in the field of drug discovery, aiding researchers in pinpointing and formulating innovative, potent, and safe pharmaceuticals [28]. The data presented in Table S3 demonstrate that all 22 compounds met the criteria outlined by Lipinski’s rules, including a molecular weight below 500 amu, less than 10 H-bond donors, fewer than five H-bond acceptors, and a lipophilicity value under 5. Additionally, they all achieved an Abbott oral bioavailability score of 0.5 or higher.

**Figure 2:**
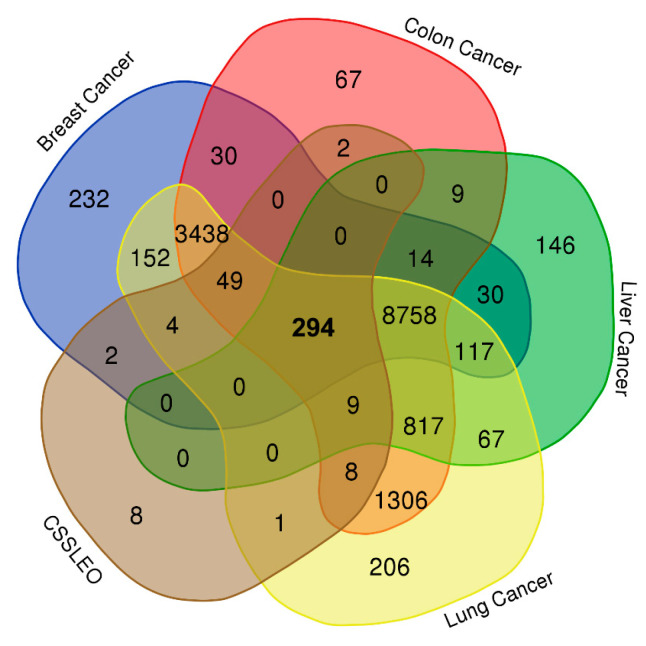
Overlapping molecular targets between breast, colon, liver, and lung cancers and CSSLEO bioactive compounds. The central bold value (294) indicates the number of molecular targets shared across all five categories. CSSLEO: *Cupressus sempervirens* ‘Stricta’ leaf essential oil.

The molecular targets linked to the bioactive components of CSSLEO were identified using the SwissTargetPrediction and TargetNet databases, resulting in a total of 377 unique targets (Table S4). Cancer-related molecular targets were then identified for breast, colon, liver, and lung cancers using the OMIM, GeneCards, and DisGeNeT databases. This process yielded 13,120 targets for breast cancer (Table S5), 14,801 targets for colon cancer (Table S6), 10,261 targets for liver cancer (Table S7), and 15,226 targets for lung cancer (Table S8). Among these, 294 targets (Table S9) overlapped with the 377 targets associated with CSSLEO bioactive compounds (Figure 2).

#### 2.3.2. Examination of Hub Genes Using Protein–Protein Interactions and Topological Analysis

**Figure 3:**
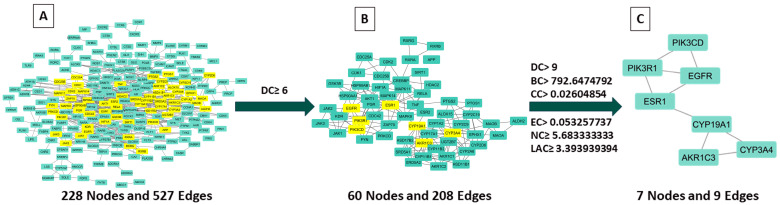
Core targets identified in PPI network analysis: (**A**) A PPI network comprising 294 protein targets; (**B**) initial selection based on exceeding twice the median value of degree centrality across 228 nodes; (**C**) subsequent refinement with centrality values surpassing the median of degree, betweenness, closeness, eigenvector, network, and local average connectivity-based method centralities for 60 nodes.

The STRING database was utilized to import the overlapping targets and create a PPI network to investigate the therapeutic mechanism of CSSLEO in combating cancer. To ensure a reliable network, disconnected nodes were eliminated at a high confidence level of 0.9, resulting in a network comprising 228 nodes and 527 edges (Figure 3A). Subsequently, the network was analyzed using Cytoscape 3.10.2. To evaluate the importance of each node, scores were generated using the CytoNCA tool, which employs various network centrality measures including degree centrality (DC), betweenness centrality (BC), closeness centrality (CC), Eigenvector centrality (EC), network centrality (NC), and local average connectivity-based method (LAC). These scores are presented in Table S10. The targets were filtered based on the median values of their scores, with two separate screenings conducted. In the first screening, a threshold of DC ≥ 6 was applied, resulting in a subset of 60 nodes and 208 edges, as depicted in Figure 3B and Table S11. Following this, a second screening was carried out to further narrow down the targets. The thresholds used in this screening were DC ≥ 9, BC ≥ 792.6474792, CC ≥ 0.02604854, EC ≥ 0.053257737, NC ≥ 5.683333333, and LAC ≥ 3.393939394. As a result, the second screening yielded seven nodes and nine edges, as shown in Figure 3C. The topological characteristics of these seven core targets are detailed in Table 5.

#### 2.3.3. Key CSSLEO Compounds Associated with Cancer Targets

**Figure 4:**
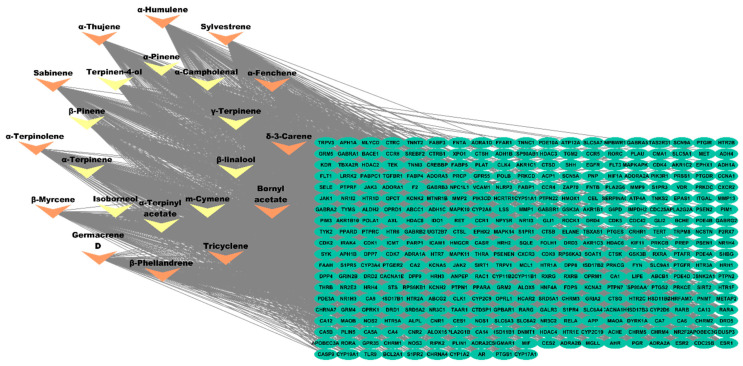
Compound-target network. The key components of CSSLEO with high degree scores are represented by yellow arrows, while the components of CSSLEO with low degree scores are represented by orange arrows. The green ovals symbolize the predictive targets of CSSLEO.

An investigation was conducted to analyze the relationship between CSSLEO compounds and their targets in cancer treatment, which was then visualized through the creation of a compound-target network using Cytoscape 3.10.2 (Figure 4). The cytoNCA plug-in was utilized to identify the key active constituents among the compounds by applying the degree centrality algorithm to filter out the most significant components. The CSSLEO compounds are organized in Table S12 based on their degree value, with ten key components identified as having degree values exceeding the median (DC > 133).

#### 2.3.4. Pathway Enrichment Analysis

**Figure 5:**
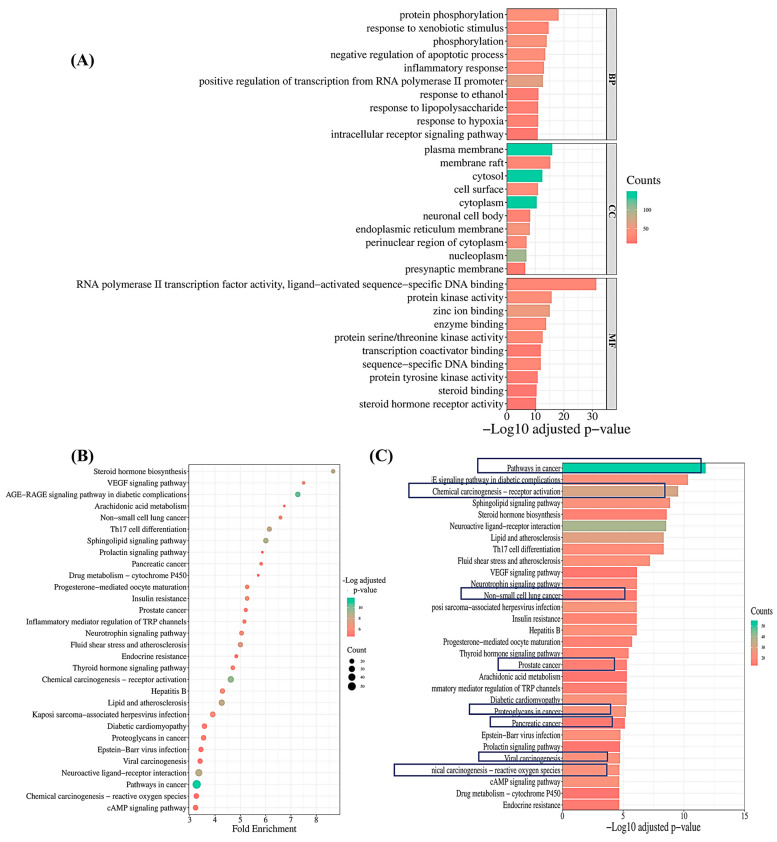
Enrichment analysis. (**A**) Key processes for GO categories. (**B**,**C**) Bubble chart and bar plot of key KEGG pathways. In (**C**), pathways surrounded by boxes indicate those particularly relevant to cancer.

In order to uncover the impact of CSSLEO on cancer treatment, gene ontology (GO) enrichment analyses were carried out on 294 common targets, resulting in the identification of a total of 489 terms across GO categories, including 319 BPs, 55 CCs, and 115 MFs. These terms were selected based on an adjusted *p*-value of less than 0.05, as indicated in Tables S13–S15, with the top ten enriched GO terms visually presented in Figure 5A. Notable BPs enriched in the analysis include pathways such as protein phosphorylation, response to xenobiotic stimulus, phosphorylation, and negative regulation of apoptotic process. Additionally, CCs like the plasma membrane, membrane raft, cytosol, and cell surface were found to be highly enriched in the study. The MFs associated with key therapeutic targets are diverse, encompassing functions like RNA polymerase II transcription factor activity, ligand-activated sequence-specific DNA binding, protein kinase activity, zinc ion binding, and enzyme binding.

Kyoto Encyclopaedia of Genes and Genomes (KEGG) pathway analysis revealed significant enrichment in 132 signaling pathways among overlapping targets, with a threshold of adjusted *p*-value < 0.05 (Table S16). The top thirty pathways with the highest negative log10-adjusted *p*-values were selected, highlighting key pathways relevant to cancer, such as pathways in cancer, chemical carcinogenesis-receptor activation, non-small-cell lung cancer, prostate cancer, proteoglycans in cancer, pancreatic cancer, viral carcinogenesis, and chemical carcinogenesis-reactive oxygen species (Figure 5B,C).

These findings shed light on the multifaceted mechanisms through which CSSLEO can contribute to cancer treatment.

#### 2.3.5. In Silico Analysis of Hub Gene Expression and Prognostic Significance

**Figure 6:**
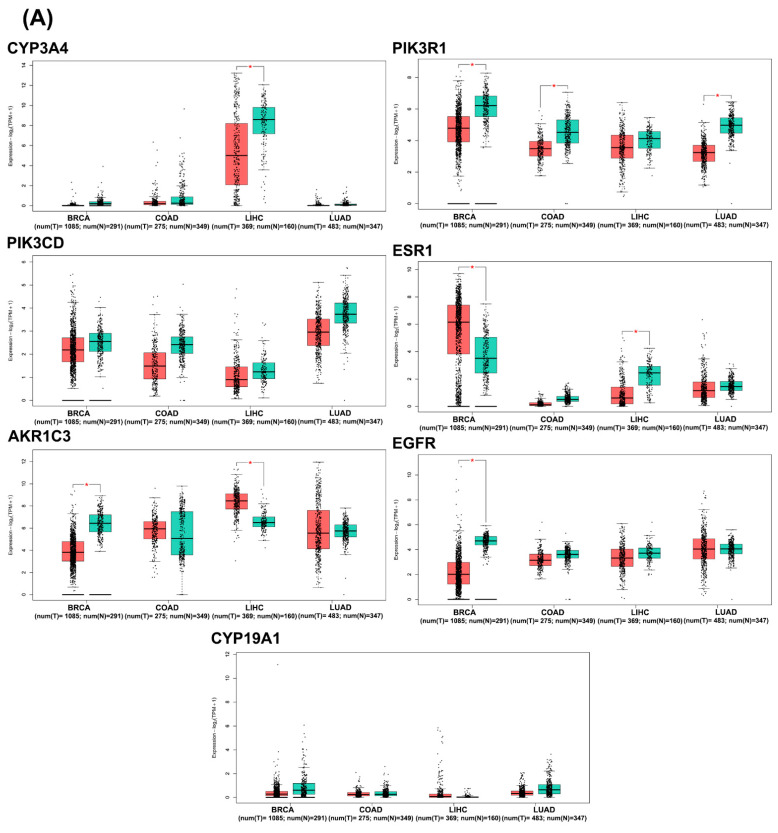

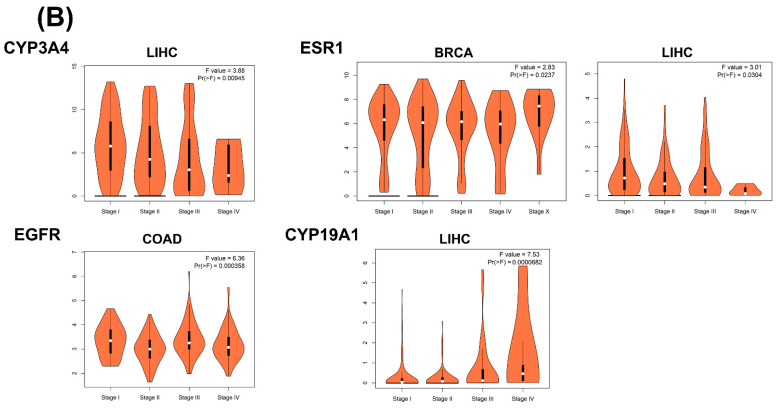
Validation of mRNA expression levels and stage plot analysis of hub genes. (**A**) The box plot illustrates the mRNA expression levels of various hub genes. In the plot, orange represents tumor tissue, while green corresponds to normal tissue (* *p* < 0.05). (**B**) The violin plots display the significant expression of hub genes across different pathological stages of tumors, as analyzed using the GEPIA2 database.

In silico analysis of hub gene expression and prognostic significance was conducted using publicly available datasets through the Gene Expression Profiling Interactive Analysis webserver (GEPIA2). Box plot analysis of the seven core target genes in breast invasive carcinoma (BRCA), colon adenocarcinoma (COAD), liver hepatocellular carcinoma (LIHC), and lung adenocarcinoma (LUAD), along with their corresponding normal tissues, revealed significant differential expression patterns (Figure 6A). Notably, PIK3R1, AKR1C3, and EGFR mRNAs were significantly downregulated in breast cancer tissue, while ESR1 was significantly upregulated. PIK3R1 also exhibited significant downregulation in colon and lung cancer tissues. In liver cancer tissues, CYP3A4 was significantly downregulated, whereas AKR1C3 was significantly upregulated.

Stage plot analysis, visualized through violin plots (Figure 6B), elucidated the mRNA expression levels of the hub targets across various stages of BRCA, COAD, LIHC, and LUAD. Statistically significant variations (*p* < 0.05) were observed in the expression levels of CYP3A, ESR1, and CYP19A1 across different stages of liver cancer. Additionally, ESR1 demonstrated significant alterations in breast cancer stages, while EGFR showed significant changes in colon cancer stages.

**Figure 7:**
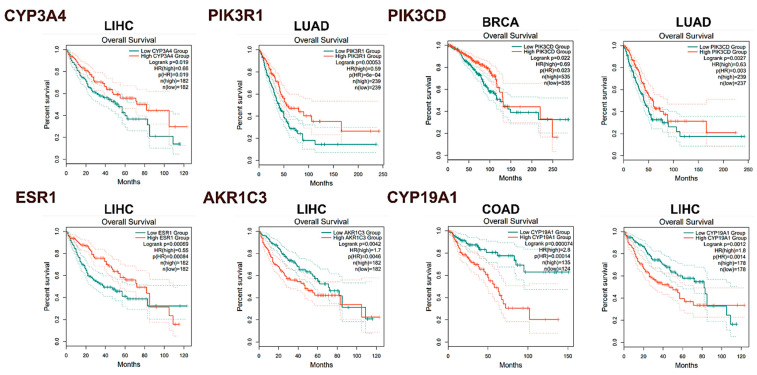
Overall survival associated with hub genes. The solid orange line signifies a group with high expression, while the solid green line represents a group with low expression. The curves were generated using the GEPIA2 database.

Kaplan–Meier survival analysis (Figure 7) revealed significant correlations between gene expression levels and overall survival (OS) across various cancer types. In BRCA patients, decreased levels of PIK3CD (HR = 0.69, log-rank *p* = 0.022) were associated with poorer prognosis. Elevated expression of CYP19A1 (HR = 2.8, log-rank *p* = 0.000074) in COAD tissues correlated with unfavorable survival outcomes. In LIHC patients, low levels of CYP3A4 (HR = 0.66, log-rank *p* = 0.019) and ESR1 (HR = 0.55, log-rank *p* = 0.00069) were linked to unfavorable OS, while increased expression of AKR1C3 (HR = 1.7, log-rank *p* = 0.0042) and CYP19A1 (HR = 1.8, log-rank *p* = 0.0012) also correlated with poorer survival. Furthermore, reduced expression of PIK3R1 (HR = 0.59, log-rank *p* = 0.00053) in LUAD patients was significantly associated with worse OS. These findings provide insights into hub gene expression patterns and potential prognostic value across multiple cancer types. However, experimental validation is needed to confirm causal relationships and functional significance in cancer progression.

The immunohistochemical findings presented in the Human Protein Atlas (HPA) database (Figure S3) indicated elevated levels of PIK3R1, ESR1, and AKR1C3 in breast cancer tissues as opposed to normal tissues. Conversely, CYP3A4 showed increased expression in normal colon tissues compared to cancerous ones. Liver cancer tissues displayed a higher expression of PIK3R1 in comparison to normal liver tissues. Furthermore, lung cancer tissues exhibited higher expression levels of PIK3R1, AKR1C3, and EGFR when compared to normal lung tissues.

### 2.4. Molecular Docking

**Figure 8:**
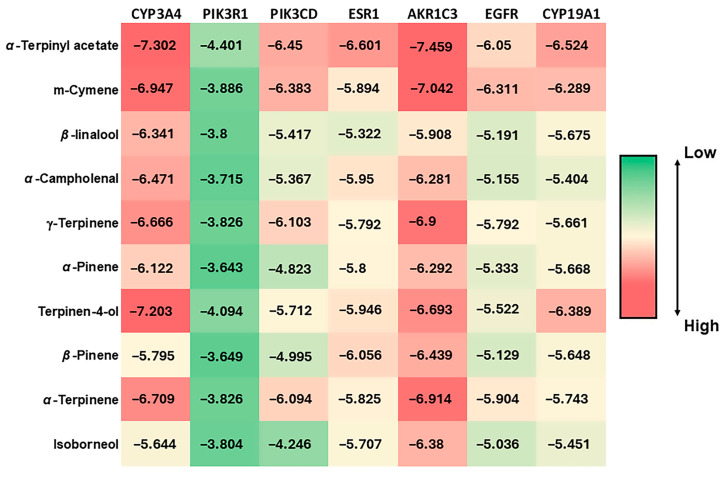
Heatmap visualizing the binding energies between key protein targets and CSSLEO phytochemicals. The Y-axis represents the phytochemicals used as ligands in the molecular docking experiment, while the X-axis represents the proteins.

A molecular docking analysis was performed using AutoDock Vina software v.1.1.2 to evaluate the binding affinity of CSSLEO compounds to key target proteins associated with cancer pathogenesis. The analysis focused on the top ten CSSLEO compounds, namely *α*-terpinyl acetate, m-cymene, *β*-linalool, *α*-campholenal, *γ*-terpinene, *α*-pinene, terpinen-4-ol, *β*-pinene, *α*-terpinene, and isoborneol (Table S12), as well as the top seven key targets: CYP3A4, PIK3R1, PIK3CD, ESR1, AKR1C3, EGFR, and CYP19A1 (Table 5). The ligand molecules were docked within a designated grid box generated around the active site of each protein. The results of the docking analysis, including the binding energies between CSSLEO phytochemicals and key proteins, are presented in the heatmap on Figure 8. A lower binding energy score indicates a stronger ligand–receptor association, with a score below −5 kcal/mol indicating a high binding affinity [29]. Figure S4A–F illustrate the top interaction complex for each protein, with the highest docking score below −5 kcal/mol.

The results indicated that the key phytochemicals derived from CSSLEO demonstrated favorable affinities for CYP3A4, with the most significant outcome observed for α-terpinyl acetate. This particular compound exhibited a docking score of −7.302 kcal/mol and participated in a total of six intermolecular interactions (Figure S4A). These interactions consisted of two alkyl bonds with ILE184, three pi-alkyl bonds with PHE271 and PHE447, and a conventional hydrogen bond with CYS442. However, the constituents of CSSLEO displayed weak affinities towards PIK3R1. On the other hand, *α*-terpinyl acetate, m-cymene, *β*-linalool, *α*-campholenal, *γ*-terpinene, terpinen-4-ol, and *α*-terpinene exhibited favorable interactions with PIK3CD, with the highest score achieved by α-terpinyl acetate (−6.45 kcal/mol) where it engaged in a total of eight intermolecular interactions, as depicted in Figure S4B. These interactions included four alkyl bonds with ILE825, VAL828, MET900, and ILE910A, along with two pi-alkyl bonds with TYR813 and PHE908, and a conventional hydrogen bond with LYS779. In the case of ESR1, all the key compounds of CSSLEO demonstrated strong bindings, with the best score achieved by α-terpinyl acetate (−6.601 kcal/mol) and a total of five intermolecular interactions, as illustrated in Figure S4C. These interactions comprised three alkyl interactions with LEU387, MET388, and LEU391, as well as one pi-alkyl bond with PHE404.

In addition, AKR1C3 strongly interacted with all the CSSLEO key phytochemicals, with *α*-terpinyl acetate exhibiting a notable binding energy score of −7.459 kcal/mol. This interaction complex involved seven intermolecular associations, including an alkyl bond with LEU54, four pi-alkyl bonds with TYR24, TYR55, TRP227, and PHE306, a pi-sigma bond with TYR24, and a conventional hydrogen bond with GLN222 (Figure S4D). On the other hand, the CSSLEO components showed good binding scores with EGFR, with m-cymene having the highest interaction score of −6.311 kcal/mol. This interaction involved ten intermolecular bindings, including five alkyl bindings with VAL765, MET766, VAL769, VAL774, and LEU833, three pi-alkyl interactions with MET766, VAL769, and ALA859, and a pi-anion bond with ASP855 (Figure S4E). Furthermore, all the key compounds effectively formed interaction complexes with CYP19A1, with *α*-terpinyl acetate having the highest binding energy of −6.524 kcal/mol. This interaction involved eight intermolecular associations, including two alkyl bonds with VAL370 and LEU477, three pi-alkyl bonds with PHE221 and TRP224, two conventional hydrogen bonds with ARG115 and MET374, and a carbon–hydrogen bond with VAL373 (Figure S4F).

## 3. Discussion

The GC–MS analysis of the leaf essential oil extracted from *Cupressus sempervirens* ‘Stricta’ (CSSLEO) grown in Egypt, for the first time, revealed a unique chemical profile. A total of 22 compounds were identified, constituting 96.01% of the oil. The major compounds were *α*-pinene (33.51%) and *δ*-3-carene (28.73%), both bicyclic monoterpene hydrocarbons, making up 62.24% of the composition. Other significant constituents included *α*-terpinolene (6.77%), *α*-terpinyl acetate (5.91%), sabinene (3.75%), and *β*-myrcene (3.74%). This composition exhibits significant variations compared to those previously documented for the essential oils of *C. sempervirens*, the Mediterranean cypress, originating from different geographic regions. For instance, Alexandrian *C. sempervirens* leaf oil primarily contains cedrol (21.29%) and *δ*-3-carene (17.85%) [30], while *C. sempervirens* L leaf oil from Cairo is rich in α-pinene (29.21%) and *δ*-3-carene (18.92%) [31]. In another investigation, it was demonstrated that the essential oil derived from the Saudi Arabian *C. sempervirens* L. aerial parts primarily contains *α*-pinene (48.6%), δ-3-carene (22.1%), limonene (4.6%), and *α*-terpinolene (4.5%) [32]. Additionally, the CSSLEO under investigation primarily comprised monoterpene hydrocarbons, contrasting with the chemically diverse *C. sempervirens* oils from other regions, which typically contain abundant monoterpene and sesquiterpene compounds [14,33]. These compositional disparities underscore the potential impact of geographical and environmental factors on essential oil biosynthesis, emphasizing the importance of comparative studies across diverse sources.

Cancer, a major contributor to worldwide mortality rates, is distinguished by unregulated cell growth and the spread of cancer cells to other parts of the body [34]. Despite advances in surgical, radiation, and chemotherapeutic treatments, the search for novel therapeutic strategies with reduced adverse effects persists. Plant-derived compounds, including EOs, are gaining attention in cancer research. Natural substances like vincristine and Taxol, as well as EOs and their components, show promise as potential anticancer agents. Studies in vitro and in vivo have demonstrated their efficacy against various tumor types. This research aims to develop more effective and less toxic cancer treatments compared to traditional chemotherapy [35].

The MTT assay revealed promising preliminary cytotoxic effects of CSSLEO on various carcinoma cell lines, particularly HepG-2 (IC_50_: 4.44 μg/mL), followed by HCT-116, A-549, and MCF-7 (IC_50_: 6.29, 6.71, and 7.20 μg/mL, respectively). The selectivity index (SI) exceeding 3 for cancer cell lines compared to normal VERO cells suggests potential cancer-specific targeting [36]. However, these results should be interpreted cautiously as initial findings requiring further investigation.

The observed effects align with previous studies on related EOs. For instance, *Juniperus phoenicea* EO demonstrated selective toxicity towards breast and colon cancer cells, attributed to high concentrations of monoterpenes, particularly *α*-pinene and *β*-pinene [37]. Similar antiproliferative effects were noted for EOs from other *Juniperus* species and *Abies* species against various cancer cell lines [38,39]. Notably, EO from the parent plant *Cupressus sempervirens* needles, with α-pinene as the primary component, showed significant cytotoxicity against NB4, HL-60, and EACC cells [31]. Additionally, *α*-terpinyl acetate from *Levisticum officinale* EO and cymene from *Thymus carmanicus* EO exhibited antiproliferative properties against specific cancer types [40,41].

While these results are encouraging, it is crucial to emphasize that the MTT assay provides only a preliminary assessment of cytotoxicity. To further explore the potential anticancer effects of CSSLEO, this research employed in silico studies to provide initial insights into the properties and potential targets of CSSLEO compounds in relation to cancer pathways. These computational analyses will offer valuable preliminary data to guide future research. However, to establish a definitive anticancer effect and fully elucidate the underlying mechanisms, additional studies will be necessary in future investigations. These may include advanced in vitro assays (e.g., apoptosis detection, cell cycle analysis), experimental investigation of molecular pathways affected by CSSLEO, evaluation of synergistic effects with established chemotherapeutic agents, and in vivo studies to assess efficacy and safety in animal models. Such comprehensive investigations, building upon the current in silico and in vitro findings, would provide a more robust foundation for potential therapeutic applications of CSSLEO in cancer treatment.

Diabetes mellitus presents a significant public health challenge, necessitating the development of safe, cost-effective, and natural therapeutic interventions. Plant-based remedies have shown promise in diabetes management, with recent research elucidating their therapeutic properties and mechanisms of action. Plant metabolites can modulate various pathways implicated in diabetes pathogenesis, including insulin secretion, glucose production inhibition, and reduction of oxidative stress and inflammation [42,43]. An essential approach in managing diabetes is focusing on digestive enzymes that play a role in breaking down carbohydrates. α-Amylase, which is mainly present in saliva and pancreatic juice, facilitates the breakdown of polysaccharides. *α*-Glucosidase, situated in the mucosal brush border of the small intestine, aids in breaking down complex carbohydrates into forms that can be absorbed. By inhibiting these enzymes, especially α-glucosidase, it is possible to reduce the absorption of glucose after meals, thereby potentially reducing spikes in blood glucose levels [44]. Evaluation of plant metabolites for their inhibitory effects on α-glucosidase and *α*-amylase provides valuable insights into their potential as natural antidiabetic agents. This approach offers a promising avenue for identifying novel, plant-derived therapeutic strategies for diabetes management [43].

CSSLEO demonstrated dose-dependent in vitro inhibitory effects on *α*-glucosidase and α-amylase, with more pronounced inhibition of *α*-amylase. This differential inhibition profile suggests a selective mechanism of action, potentially influencing postprandial glucose absorption. The effects are hypothesized to result from monoterpenes and sesquiterpenes interacting with enzyme active sites [43]. While these findings align with interest in natural products for metabolic disorder management, it is crucial to emphasize that they represent only in vitro enzymatic inhibition. Further research, particularly in vivo studies, is necessary to elucidate the physiological implications and determine potential therapeutic applications. Future investigations should focus on molecular interactions, effects on glucose metabolism in animal models, potential synergies with established treatments, and clinical safety and efficacy assessments.

Oxidative stress, an imbalance between reactive oxygen/nitrogen species and antioxidant defenses, contributes significantly to cancer and diabetes pathogenesis. In cancer, it can induce DNA damage and mutations, promoting carcinogenesis [45]. In diabetes, it disrupts enzymatic systems and enhances lipid peroxidation, leading to β-cell dysfunction [46]. These mechanisms highlight the potential therapeutic role of antioxidants in both diseases.

The antioxidant capacity of CSSLEO was significantly lower than that of ascorbic acid across DPPH, ABTS, and FRAP assays. Its antioxidant profile aligns with certain Cupressaceae species, notably *Juniperus phoenicea* EO. The Jordanian *J. phoenicea* EO demonstrated weak antioxidant activity in DPPH scavenging assays, consistent with CSSLEO’s observed properties [37].

On the other hand, the observed weak antibacterial efficacy of CSSLEO against *Bacillus subtilis* (MIC 25 mg/mL) and *Escherichia coli* (MIC 6.25 mg/mL) suggests that while CSSLEO possesses antibacterial properties, its potency is limited compared to essential oils from other *Cupressus* species, which have been reported to exhibit significant antimicrobial activities [47,48]. The absence of antibacterial effects against *Staphylococcus aureus* and *Proteus vulgaris*, as well as the lack of antifungal activity against *Aspergillus fumigatus* and *Candida albicans*, could be attributed to several factors including chemical composition, environmental factors, extraction methods, microbial resistance, concentration, and solubility [49,50].

In silico methodologies, encompassing network pharmacology and molecular docking, were employed to elucidate the properties and potential targets of CSSLEO compounds in cancer pathways. This computational approach facilitated the generation of mechanistic hypotheses and prioritization of compounds for subsequent experimental validation, providing a rational framework for investigating CSSLEO’s anticancer potential.

An ADME study of the 22 identified CSSLEO components demonstrated adherence to Lipinski’s rules of five, indicating favorable drug-likeness and potential for development as anticancer agents. The compounds achieved Abbott oral bioavailability scores ≥0.5, suggesting effective oral administration potential. While the ADME study suggests favorable properties, the challenge of CSSLEO’s water-insolubility is acknowledged as a crucial aspect affecting bioavailability. Future studies will investigate advanced techniques, including encapsulation and targeted delivery mechanisms, to optimize bioavailability and pharmacokinetic profiles of CSSLEO. These studies will focus on enhancing ADME parameters during in vivo evaluations, while prioritizing safety considerations. This approach aims to address a critical aspect in the potential therapeutic applications of CSSLEO.

The network pharmacology analysis identified 377 unique molecular targets associated with CSSLEO compounds, with 294 targets overlapping with those related to breast, colon, liver, and lung cancers. This multi-targeted approach aligns with the complex nature of cancer pathogenesis and the need for multi-faceted intervention strategies.

Topological analysis of the PPI network, using the STRING database and Cytoscape 3.10.2, identified seven core targets crucial in cancer pathways: CYP3A4, PIK3R1, PIK3CD, ESR1, AKR1C3, EGFR, and CYP19A1. These hub genes are widely recognized for their significant contributions to cancer biology.

Recent research has illuminated the critical roles of these seven core targets in cancer biology and their potential for therapeutic intervention. CYP3A4, involved in cancer cell metabolism and chemotherapy efficacy, has been identified as a potential target for enhancing treatment effectiveness through its inhibition [51]. PIK3R1, frequently mutated in triple-negative breast cancer, has been associated with other genetic alterations and higher tumor mutational burden, indicating its significance for targeted therapies [52,53]. PIK3CD has been implicated in the progression of various solid tumors, including colorectal, breast, and hepatocellular carcinoma, with its role in tumor growth and treatment resistance offering avenues for therapeutic interventions [54]. ESR1 mutations contribute to endocrine therapy resistance in metastatic hormone-receptor-positive breast cancer, emphasizing the need for a deeper understanding to develop more effective treatment strategies [55]. AKR1C3 has emerged as a potential biomarker and therapeutic target across multiple cancer types, impacting cancer cell proliferation and metastasis, and potentially enhancing immunotherapy response [56]. EGFR, despite being a longstanding target for cancer therapies, continues to present challenges due to acquired resistance, necessitating ongoing research into its signaling mechanisms and resistance pathways to improve treatment outcomes [57]. CYP19A1 has been identified as a key player in lipid metabolism and immune microenvironment modulation in colon cancer, with targeting strategies showing promise for enhancing immunotherapy efficacy and serving as a prognostic predictor [58].

The compound-target network analysis underscores the importance of the identified core targets, with the degree centrality algorithm highlighting key active constituents within the CSSLEO compounds. These widespread compounds are presumed to target CYP3A4, PIK3R1, PIK3CD, ESR1, AKR1C3, EGFR, and CYP19A1, which are known to mediate numerous signaling pathways involved in various cellular processes. This suggests that the anticancer effects of CSSLEO compounds may be attributed to their ability to modulate these core targets, thereby influencing multiple signaling cascades and eliciting diverse biological responses in vitro. However, further research is necessary to elucidate the precise mechanisms and specificity of these interactions in complex biological systems.

The comprehensive pathway enrichment analysis of CSSLEO targets has revealed valuable insights into potential molecular mechanisms in cancer treatment. Identification of 489 terms across GO categories, including BPs, CCs, and MFs, indicates CSSLEO’s multifaceted impact on cellular pathways. Key BPs, including protein phosphorylation and xenobiotic response, align with recent findings on kinase activity in cancer progression and drug resistance [59,60]. Negative regulation of apoptosis suggests CSSLEO’s potential influence on cell survival pathways [61,62,63]. CC enrichment in plasma membrane and membrane rafts corroborates current research on cell surface markers in cancer signaling and metastasis [64], while cytosolic enrichment implicates intracellular processes like lysosomal activity [65]. Diverse MFs, particularly RNA polymerase II transcription factor activity and ligand-activated sequence-specific DNA binding, reflect the complexity of gene regulation in cancer, consistent with recent studies on non-coding RNAs and chromatin remodeling in oncogenesis [66,67].

KEGG pathway analysis reinforces these findings, highlighting significant enrichment in cancer-related pathways and suggesting intricate interactions between extracellular matrix components and cell surface receptors in tumor progression [68,69]. Overall, this enrichment study indicates CSSLEO’s broad spectrum of cellular targets, providing a foundation for future investigations into its molecular mechanisms in cancer treatment.

The differential expression of PIK3R1, AKR1C3, EGFR, ESR1, PIK3CD, CYP3A4, and CYP19A1 across various cancer types and stages illuminates their potential as biomarkers for cancer progression and prognosis. GEPIA2 database analysis revealed significant downregulation of PIK3R1, AKR1C3, and EGFR mRNAs in breast cancer tissue, with ESR1 upregulation, pointing to a complex interplay in tumorigenesis and treatment response [53]. The stage plot analysis further highlights the dynamic nature of these genes across different cancer stages, particularly in liver cancer, where CYP3A4, ESR1, and CYP19A1 showed significant changes. This suggests that these genes may be involved in the progression of liver cancer and could serve as stage-specific markers [70,71]. Kaplan–Meier survival analysis correlated gene expression levels with overall survival, associating decreased PIK3CD levels with worse prognosis in breast cancer and elevated CYP19A1 expression with poorer survival in colon cancer. These findings are consistent with recent studies that have identified PIK3CD and CYP19A1 as key players in cancer progression and potential therapeutic targets [58,72,73]. Immunohistochemical data from the HPA database supported these findings, showing elevated PIK3R1, ESR1, and AKR1C3 levels in breast cancer tissues, aligning with their proposed roles in cancer cell proliferation and hormone regulation [74,75,76]. Conversely, increased CYP3A4 expression in normal colon tissues compared to cancerous ones suggests a protective role against tumorigenesis [77]. These findings collectively provide valuable insights into the differential expression patterns, stage-specific changes, prognostic significance, and protein-level alterations of the identified hub genes across multiple cancer types, laying the groundwork for further functional studies and potential therapeutic interventions.

The molecular docking analysis using AutoDock Vina has provided preliminary insights into the binding affinities of CSSLEO compounds to selected cancer-related proteins. While the observed binding energies for compounds such as α-terpinyl acetate suggest potential interactions with targets involved in drug metabolism, cell signaling, and hormone regulation, it is crucial to interpret these results with critical scrutiny.

The computational analysis indicated interactions between CSSLEO compounds and proteins involved in various cellular processes, including drug metabolism (CYP3A4) [51], cell signaling (PIK3CD, AKR1C3) [56,78], hormone regulation (ESR1) [79], and growth factor signaling (EGFR) [80]. However, the widespread nature of these phytochemicals and their potential interactions with multiple proteins necessitate a thorough evaluation of their specificity and potential off-target effects.

It is noteworthy that compounds like α-pinene, prevalent in many essential oils including CSSLEO, are classified as both pan-assay interference compounds (PAINS) and invalid metabolic panaceas (IMPS). This classification indicates such a compound’s tendency for non-specific interactions across various biological assays, potentially leading to false positive results and overestimated therapeutic potential. Such promiscuous binding behavior underscores the need for careful validation of any observed effects in molecular docking studies [81,82,83].

Ultimately, the molecular docking results suggest potential interactions between CSSLEO compounds and cancer-related proteins, but require experimental validation to confirm their pharmacological relevance. These computational findings serve as hypotheses rather than definitive evidence of therapeutic efficacy. Transitioning from in silico observations to clinically viable anticancer agents necessitates extensive in vitro and in vivo studies to evaluate pharmacokinetics, pharmacodynamics, and safety profiles.

Overall, this preliminary investigation into CSSLEO’s chemical composition and biological activities provides a foundation for understanding its therapeutic potential. The integration of GC–MS analysis, in vitro assays, and in silico studies offers initial insights into CSSLEO’s bioactive properties. These findings serve as a crucial precursor to more advanced research, bridging traditional knowledge with modern analytical techniques. Further investigation is essential to fully elucidate CSSLEO’s therapeutic potential and advance natural product research in this domain.

## 4. Materials and Methods

### 4.1. Chemicals and Reagents

#### 4.1.1. For Cytotoxic Assay

The experimental design incorporated several mammalian cell lines procured from the American Type Culture Collection (ATCC, Rockville, MD, USA). These included four carcinoma cell lines: MCF-7, HCT-116, HepG-2, and A-549, along with the Vero cell line.

For the investigation procedures, various chemicals and reagents were employed. Sigma (St. Louis, MO, USA) supplied dimethyl sulfoxide (DMSO), MTT reagent, and trypan blue dye. Lonza Bioscience (Basel, Switzerland) provided 0.25% Trypsin-EDTA solution, fetal bovine serum (FBS), HEPES buffer solution, RPMI-1640 culture medium, gentamycin, and L-glutamine.

#### 4.1.2. For Antioxidant and Antidiabetic Assay

The enzymes porcine *α*-amylase and *β*-glucosidase, along with the compounds 2,2-diphenyl-1-picrylhydrazyl (DPPH), 2,2′-azino-bis(3-ethylbenzothiazoline-6-sulfonic acid) diammonium salt (ABTS), *p*-nitrophenyl-alpha-D-glucopyranoside (pNPG), and acarbose were obtained from Sigma-Aldrich Corporation (St. Louis, MO, USA). All additional chemicals and reagents utilized in this study were of analytical grade. Double-distilled water was employed for all experimental procedures.

### 4.2. Plant Material and Sample Preparation

**Figure 9:**
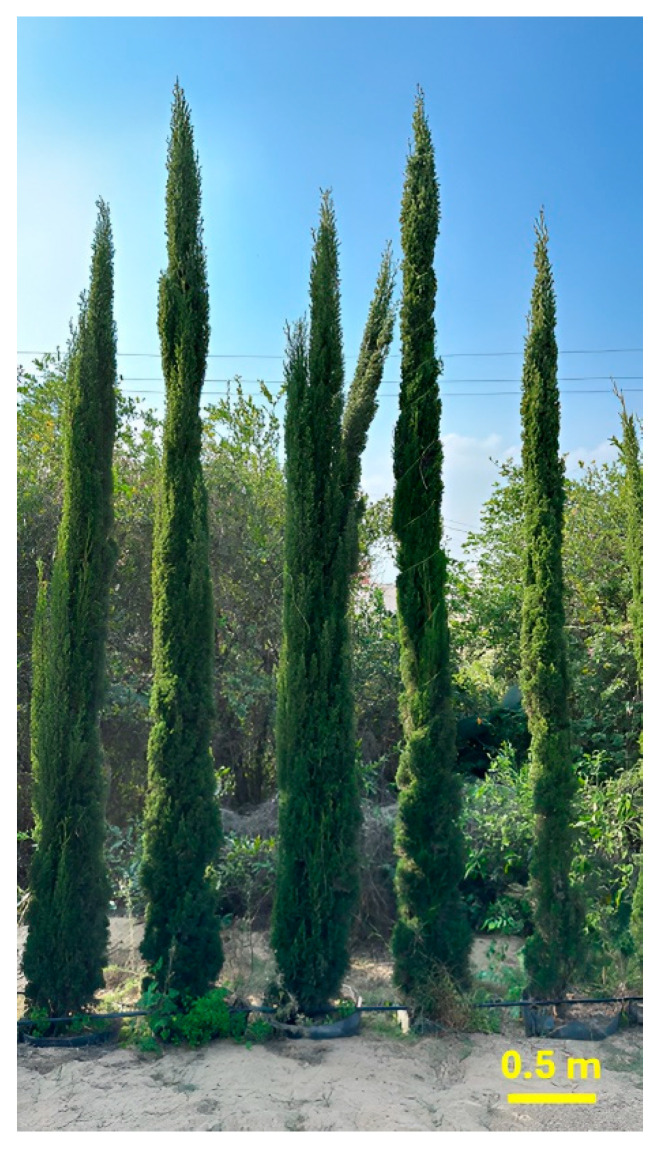
*Cupressus sempervirens* ‘Stricta’.

In February 2023, fresh leaves of *Cupressus sempervirens* ‘Stricta’ were gathered from the El-Orman Botanical Garden in Giza, Egypt (Figure 9). Taxonomic verification was performed by Eng. Therese Labib, a consultant specializing in plant taxonomy at the Egyptian Ministry of Agriculture and previously the director of the El-Orman Botanical Garden. A plant reference material (ZU-Ph-Cog-0307) was archived within the botanical repository of the Pharmacognosy Department, Zagazig University Faculty of Pharmacy.

The essential oil of *C. sempervirens* ‘Stricta’ was extracted through a hydrodistillation process. Fresh leaves (500 g) were subjected to atmospheric pressure distillation in a Clevenger-type apparatus for 5 h at approximately 100 °C. The resulting volatile oil was subsequently dehydrated using anhydrous sodium sulphate. For preservation and to facilitate further chemical and biological analyses, the extracted oil was stored in light-resistant containers at 4 °C.

### 4.3. GC–MS Analysis of CSSLEO

The chromatographic analysis was conducted using a Shimadzu GCMS-QP2010 system (Kyoto, Japan) along with an with an Rtx-5MS fused silica capillary column (Restek, Bellefonte, PA, USA) measuring 30 m in length, with an internal diameter of 0.25 mm and a film thickness of 0.25 μm. The instrument employed a split–splitless injector. The temperature program for the column began with an isothermal phase at 45 °C for 2 min, followed by a linear increase to 300 °C at a rate of 5 °C/min, concluding with a 5 min isothermal period at 300 °C. The injector temperature was maintained at 250 °C throughout the analysis. Helium was employed as the carrier gas, flowing at a rate of 1.41 mL/min. The mass spectrometer scanned a mass range of 35 to 500 *m*/*z*, utilizing an ionization voltage of 70 eV and a filament emission current of 60 mA. The ion source temperature was set at 200 °C. Samples were diluted to 1% *v*/*v*, and 1 μL aliquots were injected using a split ratio of 1:15. Component identification was achieved by comparing the retention indices (RI) and mass spectra (MS) of the analytes with those found in various reference sources. These included the Adams library [24], NIST11/2011/EPA/NIH database, Wiley library database (10th edition), and published literature data [25,26,27]. Retention indices were calculated relative to a series of n-alkane standards (C_8_–C_28_) analyzed under identical conditions. The identified components and their relative percentages were compiled and presented in Table 1.

### 4.4. In Vitro Studies

#### 4.4.1. Assessment of Cytotoxic Activity

##### Cell Line Propagation

The cytotoxic effects of CSSLEO were assessed in vitro against a panel of cancer cell lines, including MCF-7 (breast), HCT-116 (colorectal), HepG-2 (hepatocellular), and A-549 (lung). These neoplastic cells, representing various types of carcinomas, were maintained in RPMI-1640 growth medium supplemented with 10% heat-inactivated fetal calf serum and 50 μg/mL gentamycin. The culture conditions for these cancer cell lines were standardized at 37 °C in a humidified atmosphere containing 5% CO_2_, with subculturing performed two to three times weekly. As a non-cancerous counterpart, Vero cells served as a control and were cultivated in Dulbecco’s modified Eagle’s medium (DMEM) enriched with 10% heat-inactivated fetal bovine serum, 1% L-glutamine, HEPES buffer, and 50 μg/mL gentamycin. The Vero cells were maintained under similar environmental conditions as the cancer cell lines (37 °C, humidified atmosphere, 5% CO_2_), but with a reduced subculturing frequency of once every two weeks.

##### Assessment of Cytotoxicity Using MTT-Based Cell Viability Assay

Tumor and normal Vero cells were plated at 5 × 10^4^ cells/well in 96-well tissue culture plates and incubated for 24 h. CSSLEO was then added at concentrations ranging from 0.25 to 500 μg/mL in triplicate. For each 96-well microplate, a subset of six wells was allocated as vehicle controls, containing 0.5% (*v*/*v*) DMSO. Plates were incubated at 37 °C with 5% CO_2_ for 24 h. Untreated cells served as negative controls, while cisplatin-treated cells were used as positive controls. Post-incubation, media was replaced with phenol red-free RPMI 1640, and 10 μL of 12 mM MTT solution was added to each well. After 4 h, 85 μL of media was removed, and 50 μL DMSO was added. Following thorough mixing and a 10 min incubation at 37 °C, absorbance was measured at 590 nm using a microplate reader. Viability percentage was calculated as: Viability (%) = (ODt/ODc) × 100, where ODt and ODc represent the average optical densities of treated and untreated cells, respectively. Here is a rephrased version that aims to be consistent, academic, and professional while avoiding repetitive meanings: The relationship between cell viability and drug concentration is illustrated through survival curves for each tumor cell line post-treatment. The 50% inhibitory concentration (IC_50_), defined as the drug concentration at which half of the cell population exhibits cytotoxic effects, is derived from the dose–response curve analysis. This analysis is conducted for each concentration using Graphpad Prism software (San Diego, CA, USA) [84]. For the normal cell line (Vero), the equivalent metric is denoted as CC_50_ (50% cytotoxic concentration), representing the concentration at which half of the normal cells are affected.

##### Data Analysis and Statistical Procedures

Statistical analyses were performed using Statistica software version 8.0 (StatSoft Inc., Tulsa, OK, USA). Data are presented as mean values ± standard deviation (SD) based on triplicate measurements. Significant differences among groups were determined using one-way analysis of variance (ANOVA) followed by Tukey’s post hoc test. A *p*-value < 0.05 was considered statistically significant.

##### Quantifying CSSLEO’s Cytotoxic Selectivity

The selectivity index (SI) was determined by computing the ratio of the average CC_50_ value obtained from the Vero cell line to the IC_50_ values of individual cancer cell lines, specifically MCF-7, A-549, Hep-G2, and HCT-116. This calculation provides a quantitative measure of the compound’s selective cytotoxicity towards cancer cells relative to normal cells.

#### 4.4.2. Assessment of Antioxidant Activity

The antioxidant efficacy of CSSLEO was investigated through a triad of established assays: DPPH, ABTS^●+^, and FRAP. These methodologies were implemented in accordance with protocols described by Elkomy et al. [85], Ling et al. [86], and Elaasser et al. [87], respectively. To ensure reliability, each experiment was conducted in triplicate, and the mean values were computed. Ascorbic acid was employed as a reference standard throughout the antioxidant capacity assessment.

#### 4.4.3. Assessment of Antidiabetic Activity

##### *α*-Glucosidase Inhibition Activity

The *α*-glucosidase inhibitory activity was evaluated using a spectrophotometric method employing yeast α-glucosidase and *p*-nitrophenyl-*α*-D-glucopyranoside (pNPG) as the substrate [88]. The assay procedure was as follows:

The reaction mixture comprised 0.1 mL of α-glucosidase solution (1 U/mL), 0.2 mL of CSSLEO at various concentrations (0.25–500 μg/mL, prepared in 10% DMSO), and 0.5 mL of phosphate buffer (100 mM, pH 6.8). This mixture was pre-incubated at 37 °C for 15 min. Subsequently, 0.2 mL of 5 mM pNPG was added to initiate the enzymatic reaction, followed by an additional 20 min incubation at 37 °C. The reaction was terminated by adding 0.1 M sodium carbonate (Na_2_CO_3_). Absorbance readings were taken at 405 nm using a spectrophotometer. Acarbose was used as a positive control, tested at concentrations identical to those of the plant extracts. The percentage inhibition of α-glucosidase activity was calculated using the following equation: % Inhibition = [(A405blank − A405test)/A405blank] × 100, where A405blank represents the absorbance of the control reaction (without inhibitor) at 405 nm, and A405test denotes the absorbance of the sample containing the potential inhibitor at 405 nm.

##### α-Amylase Inhibition Activity

CSSLEO solutions were prepared at concentrations ranging from 0.25 to 500 μg/mL. Initially, 100 mg of the oil was solubilized in 5 mL of 10% DMSO, and the volume was adjusted to 100 mL using a buffer solution (0.02 M Na_2_HPO_4_/NaH_2_PO_4_, 0.006 M NaCl, pH 6.9), following a previously established method [89]. A stock solution of porcine pancreatic α-amylase was prepared at 2 units/mL by dissolving 12.5 mg of the enzyme in a minimum volume of 10% DMSO and diluting to 100 mL with the buffer solution. The starch substrate solution was prepared by dissolving 1 g of corn starch in 100 mL of distilled water. The assay was conducted by combining 200 μL of the CSSLEO solution with 200 μL of the α-amylase stock solution, followed by incubation at 30 °C for 10 min. Subsequently, 200 μL of the starch solution was added, and the mixture was further incubated at 30 °C for 3 min. The reaction was terminated by the addition of 3,5-dinitro salicylic acid, and the mixture was heated in a water bath at 85–90 °C for 10 min. After cooling to room temperature, 5 mL of distilled water was added. A blank solution was prepared by substituting the CSSLEO with 200 μL of buffer solution. Acarbose was employed as a positive control. The absorbance of the samples was measured at 540 nm using a UV–Vis spectrophotometer. The α-amylase inhibitory potential was calculated using the following equation: % Inhibition = [(A540blank − A540test)/A540blank] × 100%, where A540blank and A540test represent the absorbance of the blank and test samples, respectively.

##### Statistical Analysis

The inhibitory concentration at 50% (IC_50_) values were determined through nonlinear regression analysis of the dose–response curves. This analysis was performed using GraphPad Prism software version 9.4.1 (GraphPad Software Inc., San Diego, CA, USA). The software generated dose–response curves for each tested concentration, from which IC50 values were interpolated. All experiments were conducted in triplicate to ensure reproducibility. Results are presented as mean values ± standard deviation (SD).

#### 4.4.4. Assessment of Antimicrobial Activity

##### Antifungal and Antibacterial Activities (Well Diffusion Method)

The antifungal and antibacterial properties of CSSLEO were evaluated using the well diffusion method [90]. The study assessed its efficacy against two fungal strains: *Aspergillus fumigatus* (RCMB 002008) and *Candida albicans* (RCMB 005003). Additionally, the compound’s antibacterial activity was tested against two Gram-positive bacteria, *Staphylococcus aureus* (ATCC 25923) and *Bacillus subtilis* (RCMB 015 NRRL B-543), as well as two Gram-negative bacteria, *Escherichia coli* (ATCC 25922) and *Proteus vulgaris* (RCMB 004 ATCC 13315). All microbial strains were sourced from the Regional Center for Mycology and Biotechnology at Al-Azhar University, Egypt. For fungal assays, potato dextrose agar (PDA) was used as the culture medium. The test samples were prepared by dissolving CSSLEO in dimethyl sulfoxide (DMSO) to achieve a concentration of 50 mg/mL. Ketoconazole (100 μg/mL) and Gentamycin (4 mg/mL) served as positive controls for fungi and bacteria, respectively. The assay was conducted by creating 6 mm-diameter wells in the agar plates, which were then filled with 100 μL of the sample solution. DMSO was used as a negative control. The inoculated plates were incubated at 37 °C for 24–48 h. Each assay was performed in triplicate to ensure reliability. The antimicrobial activity was quantified by measuring the diameter of the inhibition zones. Results were reported as the mean inhibition zone diameter (in mm) ± standard deviation (SD). This methodology aligns with established protocols in antimicrobial susceptibility testing [91].

##### Determination of Minimum Inhibitory Concentration (MIC)

The MIC values of the investigated oil sample were determined using the agar dilution method. The sample was initially dissolved in DMSO and subsequently subjected to serial dilution to achieve a concentration range of 0.39 to 50 mg/mL. Bacterial isolates were cultured on PDA plates, from which individual colonies were selected and suspended in saline solution. The turbidity of the bacterial suspension was adjusted to match the 0.5 McFarland standard, corresponding to approximately 5 × 10^6^ colony-forming units per milliliter (cfu/mL). This suspension was further diluted 1:10 with saline. PDA plates containing various concentrations of the serially diluted sample were prepared. Each plate was inoculated with 2 μL of the prepared bacterial suspension, resulting in a final inoculum concentration of 10^3^ cfu per spot. The inoculated plates were then incubated at 30 °C for a period of 24 to 48 h. Throughout the incubation period, the plates were monitored for signs of bacterial growth to determine the MIC values [92,93].

### 4.5. Network Pharmacology

#### 4.5.1. Evaluating the Pharmacokinetic Properties of CSSLEO Phytoconstituents

The canonical SMILES formulas of the 22 phytoconstituents that were identified through GC–MS analysis were gathered from the PubChem database (https://pubchem.ncbi.nlm.nih.gov/, accessed on 20 March 2024) or generated using ChemDraw v22.0.0.22 (PerkinElmer Informatics, Inc., Buckinghamshire, UK). Subsequently, these SMILES formulas were inputted into the SwissADME web server for drug-like property assessment, where specific screening criteria such as Lipinski’s rule [94] and a minimum Abbott oral bioavailability score above 0.5 were applied to evaluate their pharmaceutical potential.

#### 4.5.2. Identification of the Intersection Genes between Breast, Colon, Liver, and Lung Cancers and CSSLEO Bioactive Compounds

Putative targets associated with the compounds were extracted from two predictive databases: Swiss Target Prediction (STP, http://www.swisstargetprediction.ch/) [95] and TargetNet (http://targetnet.scbdd.com/) [96], accessed on 22 March 2024. To compile cancer-related targets, three comprehensive databases were utilized: Online Mendelian Inheritance in Man (OMIM, https://www.omim.org/) [97], DisGeNet (https://www.disgenet.org/search), and GeneCards (https://www.genecards.org/) [98,99], accessed on 23 March 2024. The search process for cancer-related targets was conducted separately for each cancer type, employing specific search terms such as “Breast carcinoma”, “Colon Carcinoma”, “Hepatocellular Carcinoma”, and “Lung Carcinoma”.

Following target identification, UniProt IDs and gene symbols were obtained from the UniProt database on 25 March 2024 (https://www.uniprot.org/) [100]. To maintain data integrity, duplicate targets were eliminated from the dataset.

To visualize and analyze the intersection between targets associated with the bioactive compounds and those linked to the four cancer types, a Venn diagram was generated using an online bioinformatics tool (https://bioinformatics.psb.ugent.be/webtools/Venn/, accessed on 25 March 2024).

#### 4.5.3. Constructing the Protein–Protein Interaction (PPI) Network

Following the acquisition of overlapping targets, the STRING database v12.0 (https://string-db.org/, accessed on 25 March 2024) [101] was employed to examine the protein–protein interactions (PPI). The analysis focused on the interactions with a confidence level greater than 0.9, specifically within the species *Homo sapiens*.

The resulting interaction data were then imported into Cytoscape software version 3.10.2 (NIGMS, Bethesda, MD, USA) for the purpose of visualizing the networks [102]. Within Cytoscape, the CytoNCA tool was employed to analyze the network topology parameters of the targets, including DC, BC, CC, EC, NC, and LAC [103]. The DC represents the number of edges connecting to a node, indicating its significance. The BC signifies a target’s bridging role within the network. The CC measures the proximity between a target and all other targets in the network. The EC depicts a target’s connectivity with other pivotal targets in the network. Lastly, the overall significance of a target in the network is indicated by the NC, while the level of clustering of a target in the network is quantified by the LAC [104]. This analysis aimed to identify hub networks and core targets through a two-step filtration process. The data were first processed to ensure that DC was at least 2 times the median value. A secondary screening was then done to identify core targets with DC, BC, EC, CC, NC, and LAC above or equal to their respective median values [105]. This filtering methodology allowed us to key nodes in the network with the highest importance and influence, which could potentially play a crucial role in in the progression of the disease under investigation or become potential targets for therapeutic interventions.

#### 4.5.4. Establishing the Compound-Target Interaction Network

A compound-target network was created in Cytoscape 3.10.2 to visualize the connection between the bioactive components of CSSLEO and their potential targets for cancer therapy. Within the network, candidate compounds were depicted as nodes, and the interactions between compounds and targets were represented by edges. Using the CytoHubba plugin in Cytoscape [106], essential active constituents of CSSLEO were identified by screening them based on their degree value, highlighting their significance in potential cancer treatment. Furthermore, the key components were determined by selecting the components with a degree value higher than the median.

#### 4.5.5. Enrichment Analysis of GO and KEGG Pathways

The critical therapeutic targets within the biological system and signalling pathways underwent analysis through GO and KEGG enrichment analysis. The overlapping genes were inputted into the Database for Annotation, Visualization, and Integrated Discovery (DAVID, https://david.ncifcrf.gov/, accessed on 28 March 2024) [107] to anticipate the three GO categories (molecular functions-MF, biological processes-BP, cellular components-CC) and KEGG pathways associated with overlapping genes. *Homo sapiens* (Human) was chosen as the organism for this study, and the statistical significance was determined using the Benjamini–Hochberg procedure for multiple testing correction, with a threshold of *p* < 0.05. Subsequently, the visualization analysis was carried out using the Bioinformatics Platform (http://www.bioinformatics.com.cn/en, accessed on 31 March 2024) to generate bar plots and bubble charts for further examination and interpretation.

#### 4.5.6. Computational Analysis of Gene Expression in Cancer Datasets

To further investigate the potential role of the identified core target genes in various cancers, their expression patterns and clinical significance were explored using publicly available databases. The GEPIA2 resource (http://gepia.cancer-pku.cn/, accessed on 3 April 2024) [108,109] was employed to analyze the mRNA expression levels of the seven core target genes. This analysis focused on four cancer types: BRCA, COAD, LIHC, and LUAD. Additionally, expression levels in corresponding normal tissues from the Genotype-Tissue Expression (GTEx) project were evaluated. GEPIA2 was utilized to assess the association between gene expression and clinicopathological features, specifically focusing on the pathological stage of the cancers. Data from The Cancer Genome Atlas (TCGA) project were used for this analysis.

Furthermore, the potential association between gene expression and OS was investigated using Kaplan–Meier survival analysis in GEPIA2. For each gene, patients were stratified into high and low expression groups based on the median expression level. The log-rank test was used to compare survival curves, and hazard ratios (HRs) with 95% confidence intervals were calculated. The HR value indicates the relative risk of death in the high expression group compared to the low expression group. An HR > 1 suggests that higher gene expression is associated with increased risk, while an HR < 1 suggests that higher gene expression is associated with decreased risk. Statistical significance was set at *p* < 0.05 for the log-rank test. To examine protein expression patterns of these hub genes, immunohistochemistry data from the HPA (https://www.proteinatlas.org/, accessed on 5 April 2024) were analyzed [110]. Staining intensity and patterns in cancer tissues were compared to those in corresponding normal tissues.

### 4.6. Molecular Docking Study

This study investigated the molecular interactions between 10 key active ingredients and 7 core target proteins using computational docking analysis. Three-dimensional crystal structures of the target proteins, obtained from the Protein Data Bank in 10 April 2024 (http://www.rcsb.org) [111], included CYP3A4 (PDB ID: 1TQN/2.05 Å) [112], PIK3R1 (PDB ID: 4JPS/2.20 Å) [113], PIK3CD (PDB ID: 6PYR/2.21 Å) [114], ESR1 (PDB ID: 1SJ0/1.90 Å) [115], AKR1C3 (PDB ID: 4H7C/1.97 Å) [116], EGFR (PDB ID: 5U8L/1.60 Å) [117], and CYP19A1 (PDB ID: 3S79/2.75 Å) [118]. The protein structures were prepared using Biovia Discovery Studio v21.1.0.20298 [119] and USCF Chimera 1.17.3 software [120] as previously described [121]. For CYP3A4, PIK3R1, and CYP19A1, which lacked co-crystallized ligands, the Computed Atlas for Surface Topography of Proteins server (CASTp; http://sts.bioe.uic.edu/castp/index.html) [122] was accessed on 11 April 2024, to predict active pockets and determine coordinates. The active compounds were obtained from the PubChem database and converted to dockable formats using OpenBabel 2.4.1 [123]. Molecular docking was performed using AutoDock Vina 1.1.2, with grid coordinates for each protein’s active site established through the AutoDock Vina suite in UCSF Chimera. The docking process generated 10 unique poses for each ligand–protein interaction. The details of the grid boxes, including their centers, sizes, and the amino acid residues of the active sites, can be found in Table S17. Binding affinities were assessed using docking scores (kcal/mol), with lower scores indicating stronger interactions. The best-scoring pose with minimal root mean square deviation (RMSD) value was selected for each ligand. To ensure accuracy, co-crystallized ligands were re-docked where available to validate the active sites.

The resulting molecular interactions were visualized and analyzed using Biovia Discovery Studio Visualizer v21.1.0.20298 (BIOVIA Dassault Systemes, San Diego, CA, USA).

## 5. Conclusions

In conclusion, this research integrates chemical analysis, in vitro assays, and in silico studies to investigate the diverse bioactive profile of *Cupressus sempervirens* ‘Stricta’ leaf essential oil (CSSLEO) from Egypt, and its potential therapeutic properties. While preliminary results show promise in cytotoxicity against cancer cell lines and inhibition of diabetes-related enzymes, these findings warrant further rigorous validation and critical evaluation within a broader experimental context. The identification of key molecular targets and potential mechanisms of action through computational methods offers a foundation for future research. However, extensive further investigation, including advanced in vitro and in vivo studies, is necessary to validate these initial findings and fully understand CSSLEO’s therapeutic potential. This study serves as a starting point for more targeted research into the possible applications of CSSLEO in cancer and diabetes management.

## Figures and Tables

**Table 1:** Chemical composition of *Cupressus sempervirens* ‘Stricta’ leaf essential oil.

Peak	Rt	Compound Name	Molecular Formula	Chemical Class	RI_Exp._^a^	RI_Lit_^b^	Area %	Identification ^c^
1	7.205	Tricyclene	C_10_H_16_	Tricyclic monoterpene hydrocarbon	911	902	0.17	MS, RI
2	7.370	*α*-Thujene	C_10_H_16_	Bicyclic monoterpene hydrocarbon	917	908	0.85	MS, RI
3	7.575	*α*-Pinene	C_10_H_16_	Bicyclic monoterpene hydrocarbon	923	916	33.51	MS, RI
4	7.855	*α*-Fenchene	C_10_H_16_	Bicyclic monoterpene hydrocarbon	933	929	1.50	MS, RI
5	8.615	Sabinene	C_10_H_16_	Bicyclic monoterpene hydrocarbon	958	958	3.75	MS, RI
6	8.705	*β*-Pinene	C_10_H_16_	Bicyclic monoterpene hydrocarbon	961	961	1.64	MS, RI
7	9.210	*β*-Myrcene	C_10_H_16_	Acyclic monoterpene hydrocarbon	978	978	3.74	MS, RI
8	9.825	*δ*-3-Carene	C_10_H_16_	Bicyclic monoterpene hydrocarbon	998	999	28.73	MS, RI
9	9.945	*α*-Terpinene	C_10_H_16_	Monocyclic monoterpene hydrocarbon	1002	1006	0.84	MS, RI
10	10.040	*m*-Cymene	C_10_H_14_	Aromatic monoterpene hydrocarbon	1005	1012	0.11	MS, RI
11	10.255	*β*-Phellandrene	C_10_H_16_	Monocyclic monoterpene hydrocarbon	1012	1013	1.16	MS, RI
12	10.320	Sylvestrene	C_10_H_16_	Monocyclic monoterpene hydrocarbon	1014	1018	1.99	MS, RI
13	11.200	*γ*-Terpinene	C_10_H_16_	Monocyclic monoterpene hydrocarbon	1042	1048	1.24	MS, RI
14	12.115	*α*-Terpinolene	C_10_H_16_	Monocyclic monoterpene hydrocarbon	1071	1078	6.77	MS, RI
15	12.945	*β*-linalool	C_10_H_18_O	Acyclic monoterpene alcohol	1098	1090	0.13	MS, RI
16	13.725	*α*-Campholenal	C_10_H_16_O	Monocyclic monoterpene aldehyde	1123	1125	0.19	MS, RI
17	14.525	Isoborneol	C_10_H_18_O	Bicyclic monoterpene alcohol	1149	1147	0.26	MS, RI
18	14.610	Terpinen-4-ol	C_10_H_18_O	Monocyclic monoterpene alcohol	1152	1167	2.36	MS
19	17.810	Bornyl acetate	C_12_H_20_O_2_	Bicyclic monoterpene ester	1259	1276	0.31	MS
20	19.640	*α*-Terpinyl acetate	C_12_H_20_O_2_	Monocyclic monoterpene ester	1323	1339	5.91	MS
21	22.710	*α*-Humulene	C_15_H_24_	Sesquiterpene hydrocarbon	1436	1445	0.20	MS
22	23.390	Germacrene D	C_15_H_24_	Sesquiterpene hydrocarbon	1462	1473	0.65	MS
Total identified							96.01	
Monoterpene hydrocarbons							86.00	
Oxygenated monoterpenes							9.16	
Sesquiterpene hydrocarbons							0.85	

Compounds are arranged based on their elution sequence on the Rtx-5MS GC column. ^a^ The retention index was experimentally determined on an Rtx-5MS column in comparison to C_8_–C_28_ n-alkanes. ^b^ Reported retention index. ^c^ To establish identification, the mass spectral (MS) data and retention index (RI) values of the compounds were compared with those in the Adams library [24], NIST 11 Mass Spectral Library, Wiley Registry of Mass Spectral Data 10th edition, and relevant literature [25,26,27].

**Table 2:** The cytotoxic effects of CSSLEO and cisplatin on Vero, MCF-7, HCT-116, HepG-2, and A-549 cell lines are presented based on CC_50_, IC_50_ values in μg/mL (Mean ± SD), and SI.

	Vero	MCF-7		HCT-116		HepG-2		A-549	
	CC_50_	IC_50_	SI	IC_50_	SI	IC_50_	SI	IC_50_	SI
CSSLEO	26.95 ± 1.06	7.20 ± 0.32	3.74	6.29 ± 0.29	4.35	4.44 ±0.21	6.07	6.71 ± 0.28	4.02
Cisplatin	40.68 ± 2.75	5.57 ± 0.68	7.30	2.75 ± 0.24	14.28	3.87 ± 0.55	10.52	7.68 ± 0.36	5.29

**Table 3:** Antioxidant activity of CSSLEO.

	IC_50_ ± SD (μg/mL)		
	DPPH	ABTS	FRAP
CSSLEO	212.08 ± 4.71	138.20 ± 3.69	307.35 ± 6.04
Ascorbic acid	10.19 ± 0.65	10.78 ± 0.65	20.88 ± 0.94

**Table 4:** The antimicrobial activity of CSSLEO.

Tested Sample	Inhibition Zone (IZ mm) Diameter (Mean ± SD)/Minimum Inhibitory Concentration (MIC mg/mL)											
	Fungi				Gram-Positive Bacteria				Gram-Negative Bacteria			
	*Aspergillus fumigatus* (RCMB 002008)		*Candida albicans* (RCMB 005003 (1) ATCC 10231)		*Staphylococcus aureus* (ATCC 25923)		*Bacillus subtilis* RCMB 015 (1) NRRL B-543		*Escherichia coli* ATCC 25922		*Proteus vulgaris* RCMB 004 (1) ATCC 13315	
	IZ	MIC	IZ	MIC	IZ	MIC	IZ	MIC	IZ	MIC	IZ	MIC
CSSLEO	NA	-	NA	-	NA	-	8 ± 0.56	25 ± 0.97	13 ± 0.62	6.25 ± 0.83	NA	-
Ketoconazole (100 mg/mL)	17 ± 0.36	-	20 ± 0.27	-	-	-	-	-	-	-	-	-
Gentamycin (4 mg/mL)	-	-	-	-	24 ± 0.58	-	26 ± 0.68	-	30 ± 0.73	-	25 ± 0.81	-

NA: No activity.

**Table 5:** The core protein targets pinpointed through the analysis of PPI and their key topological features (DC = degree centrality, BC = betweenness centrality, CC = closeness centrality, EC = Eigenvector centrality, NC = network centrality, and LAC = local average connectivity-based method).

Target Name	Description	DC	BC	CC	EC	NC	LAC
CYP3A4	Cytochrome P450 3A4	22	5123.8018	0.026203394	0.328590542	14.08333333	4.363636364
PIK3R1	Phosphoinositide-3-kinase regulatory subunit 1	19	4439.497006	0.026478479	0.062474731	11.61941474	3.789473684
PIK3CD	Phosphatidylinositol 4,5-bisphosphate 3-kinase catalytic subunit delta isoform	18	2712.974904	0.026340218	0.054360334	11.06023046	3.888888889
ESR1	Estrogen receptor	18	7909.706627	0.026640066	0.098252818	9.888965283	4.222222222
AKR1C3	Aldo-keto reductase family 1 member C3	16	2055.232626	0.026053024	0.310182363	12.1260101	5.875
EGFR	Epidermal growth factor receptor	15	3023.4256	0.026383078	0.058514997	6.031962482	3.466666667
CYP19A1	Cytochrome P450 19A1 (Human aromatase)	12	6596.306973	0.026364692	0.249898016	7.346969697	5

## Data Availability

All data and materials used are available in the manuscript.

## Supplementary Materials

The following supporting information can be downloaded at: https://www.mdpi.com/article/10.3390/ph17081019/s1, Figure S1: Chemical structures of the identified components in Cupressus sempervirens ‘stricta’ leaf essential oil; Figure S2: The in vitro cytotoxic effects of Cupressus sempervirens ‘Stricta’ leaf essential oil and cisplatin on various carcinoma cell lines; Figure S3: The immunohistochemical images of the protein expression of the core targets in breast, colon, liver, lung cancers and their respective normal tissues obtained from the Human Protein Atlas (HPA) database; Figure S4: Illustrations of molecular docking results presented in both three-dimensional and two-dimensional formats; Table S1: Inhibition effects of CSSLEO on α-Glucosidase enzyme activity; Table S2: Inhibition effects of CSSLEO on α-amylase enzyme activity; Table S3: Pharmacokinetics and the drug-likeness properties of CSSLEO constituents; Table S4: Molecular targets of CSSLEO compounds; Table S5: Molecular targets associated with breast cancer; Table S6: Molecular targets associated with colon cancer; Table S7: Molecular targets associated with liver cancer; Table S8: Molecular targets associated with lung cancer; Table S9: Overlapping targets between CSSLEO and breast, colon, liver and lung cancers; Table S10: The topological features of the PPI nodes; Table S11: The protein targets obtained through the first filtration step and their topological features; Table S12: The identified compounds of CSSLEO ranked by the degree scores; Table S13: Details of GO analysis pertaining to biological processes; Table S14: Details of GO analysis pertaining to cellular components; Table S15: Details of GO analysis pertaining to molecular functions; Table S16: Details of KEGGs pathway analysis; Table S17: Target proteins, the corresponding grid coordinates, and amino acid residues of the active sites.
