# Supplementary material for: Unveiling the Bioactive Efficacy of Cupressus sempervirens ‘Stricta’ Essential Oil: Composition, In Vitro Activities, and In Silico Analyses
Source: Pharmaceuticals (Basel). 2024 Aug 2;17(8):1019. doi: 10.3390/ph17081019 (PMC11357629; doi:10.3390/ph17081019)
Supplement: Supplementary file 1 [file pharmaceuticals-17-01019-s001.zip › Figure S1-S4.pdf]

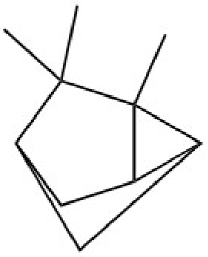

**Tricyclene**

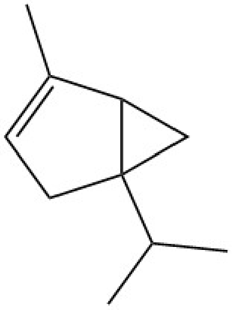

***α*-Thujene**

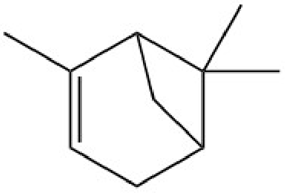

***α*-Pinene**

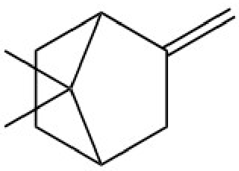

***α*-Fenchene**

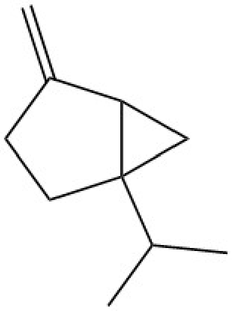

**Sabinene**

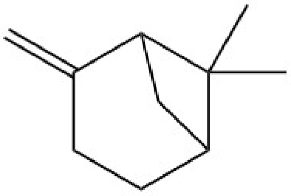

***β*-Pinene**

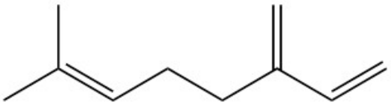

***β*-Myrcene**

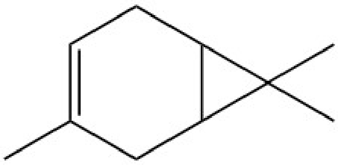

***δ*-3-Carene**

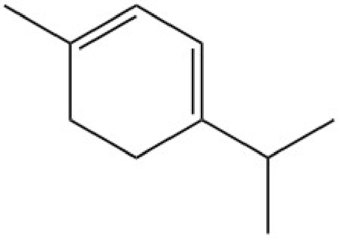

***α*-Terpinene**

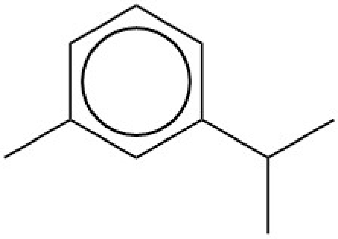

***m*-Cymene**

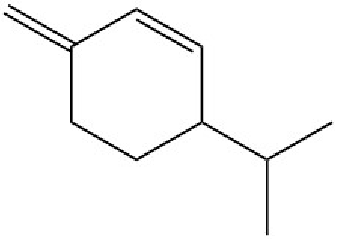

***β*-Phellandrene**

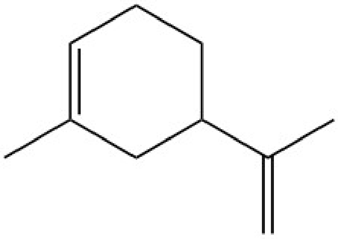

**Sylvestrene**

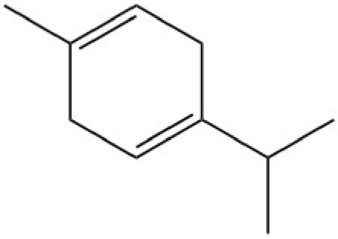

***γ*-Terpinene**

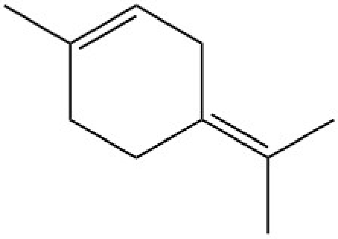

***α*-Terpinolene**

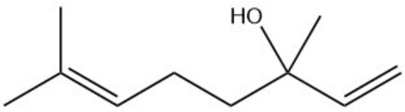


***β*-linalool**

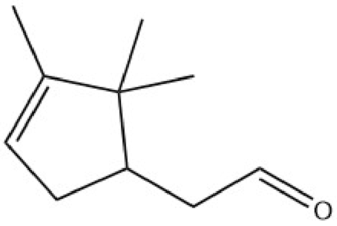


***α*-Campholenal**

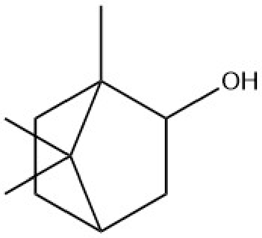


**Isoborneol**

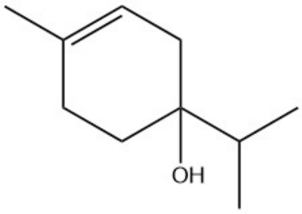


**Terpinen-4-ol**

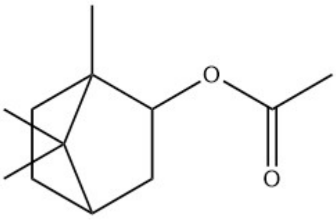


**Bornyl acetate**

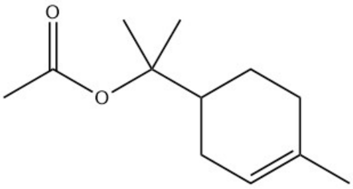


**$\alpha$-Terpinyl acetate**

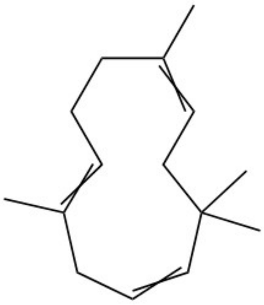

**$\alpha$-Humulene**

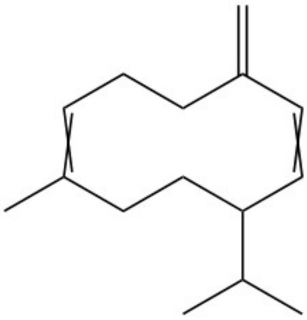

**Germacrene D**

**Figure S1.** Chemical structures of the identified components in *Cupressus sempervirens* 'stricta' leaf essential oil.

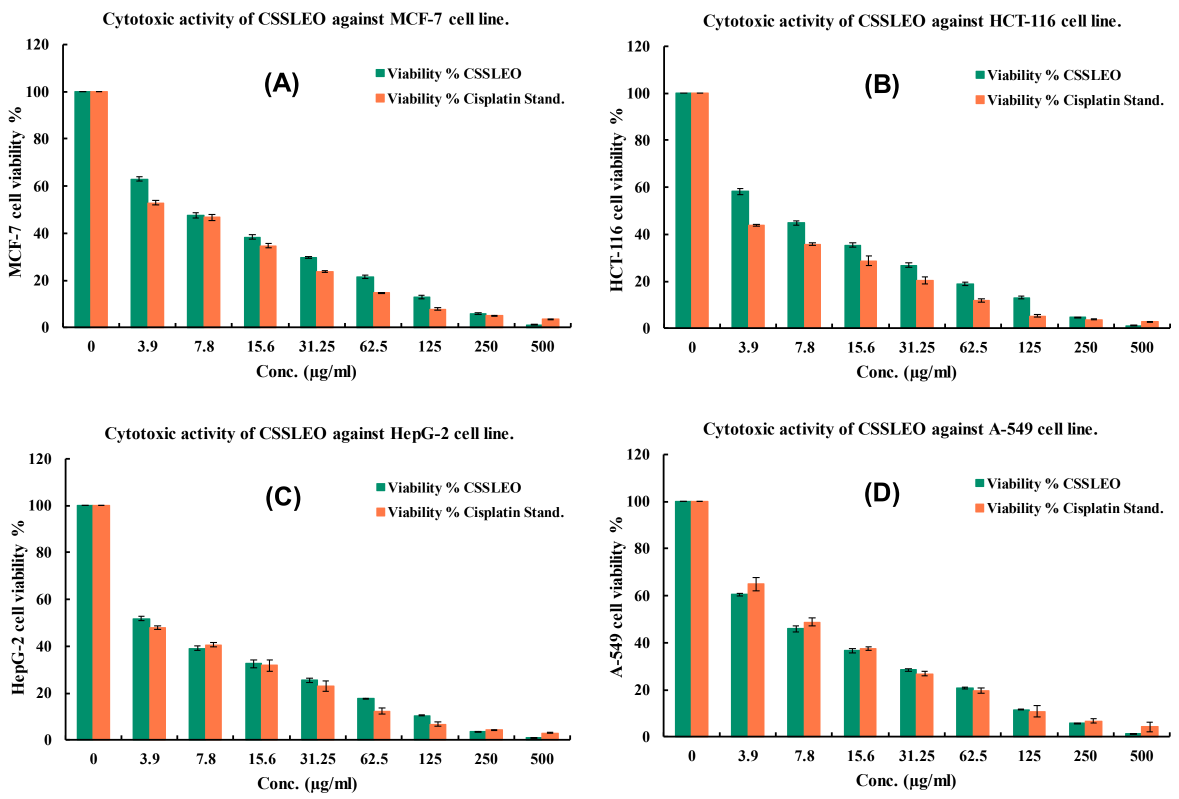


**Figure S2.** The *in vitro* cytotoxic effects of *Cupressus sempervirens* 'Stricta' leaf essential oil and cisplatin on various carcinoma cell lines including (A) human breast (MCF-7), (B) colon (HCT-116), (C) hepatocellular (HepG-2), and (D) lung (A-549) carcinoma cell lines.

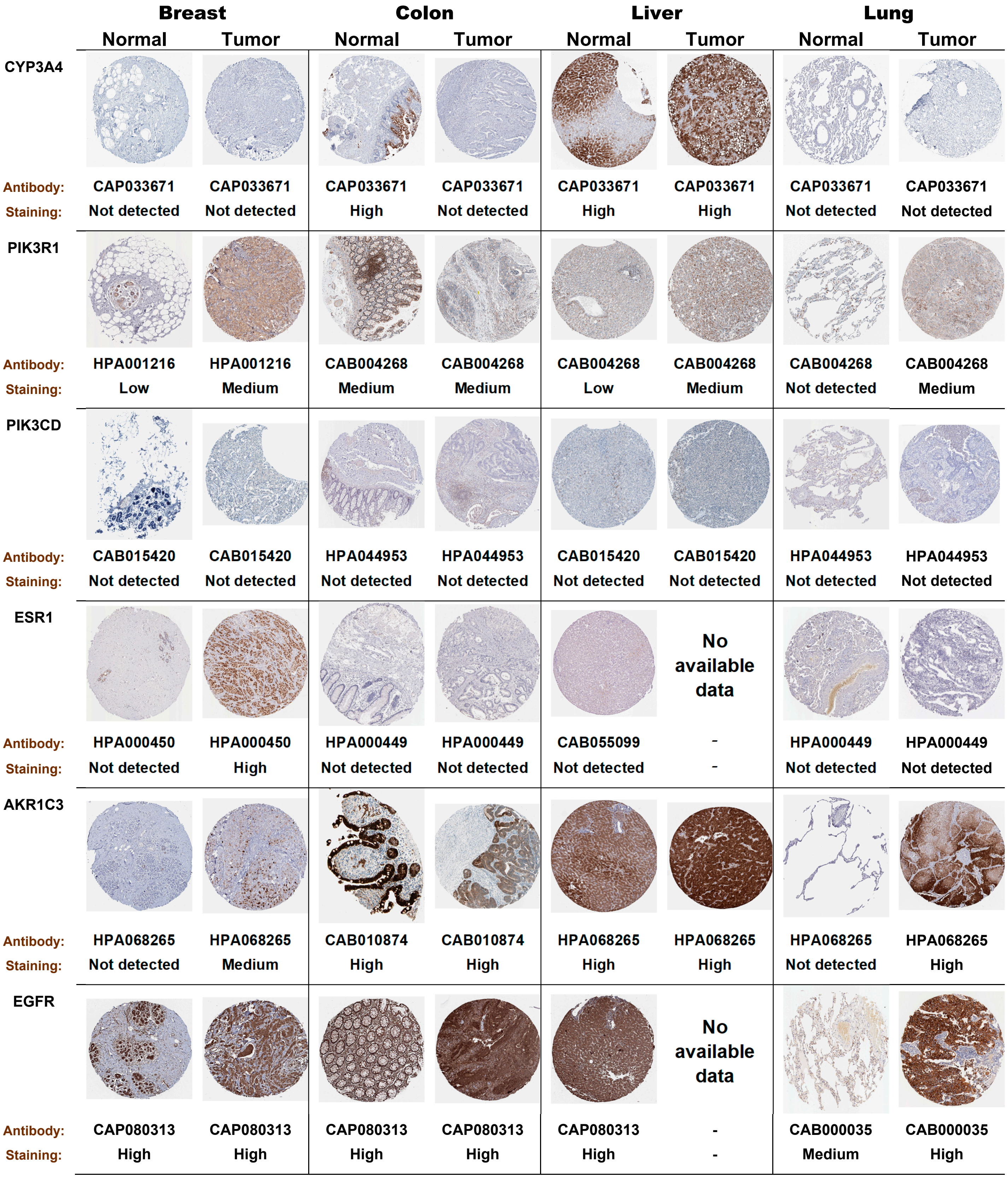

Breast
Colon
Liver
Lung
Normal
Tumor
Normal
Tumor
Normal
Tumor
Normal
Tumor
CYP3A4
Antibody:
CAP033671
CAP033671
CAP033671
CAP033671
CAP033671
CAP033671
CAP033671
CAP033671
Staining:
Not detected
Not detected
High
Not detected
High
High
Not detected
Not detected
PIK3R1
Antibody:
HPA001216
HPA001216
CAB004268
CAB004268
CAB004268
CAB004268
CAB004268
CAB004268
Staining:
Low
Medium
Medium
Medium
Low
Medium
Not detected
Medium
PIK3CD
Antibody:
CAB015420
CAB015420
HPA044953
HPA044953
CAB015420
CAB015420
HPA044953
HPA044953
Staining:
Not detected
Not detected
Not detected
Not detected
Not detected
Not detected
Not detected
Not detected
ESR1
No available data
Antibody:
HPA000450
HPA000450
HPA000449
HPA000449
CAB055099
-
HPA000449
HPA000449
Staining:
Not detected
High
Not detected
Not detected
Not detected
-
Not detected
Not detected
AKR1C3
Antibody:
HPA068265
HPA068265
CAB010874
CAB010874
HPA068265
HPA068265
HPA068265
HPA068265
Staining:
Not detected
Medium
High
High
High
High
Not detected
High
EGFR
No available data
Antibody:
CAP080313
CAP080313
CAP080313
CAP080313
CAP080313
-
CAB000035
CAB000035
Staining:
High
High
High
High
High
-
Medium
High

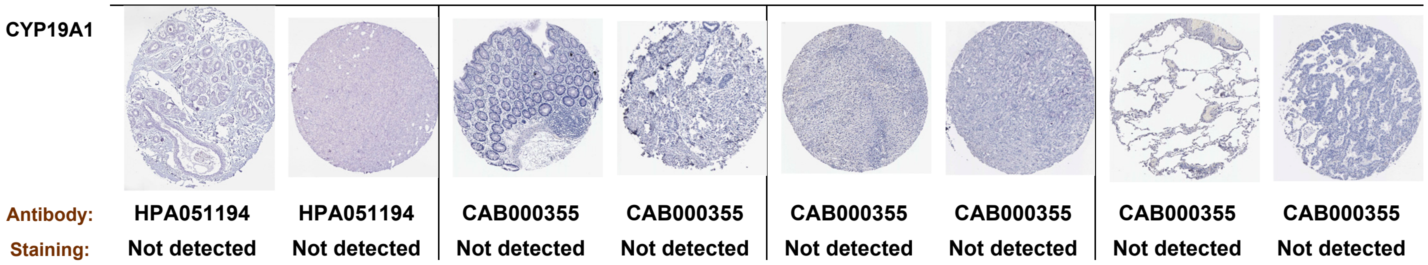


**Figure S3.** The immunohistochemical images of the protein expression of the core targets in breast, colon, liver, lung cancers and their respective normal tissues obtained from the Human Protein Atlas (HPA) database.

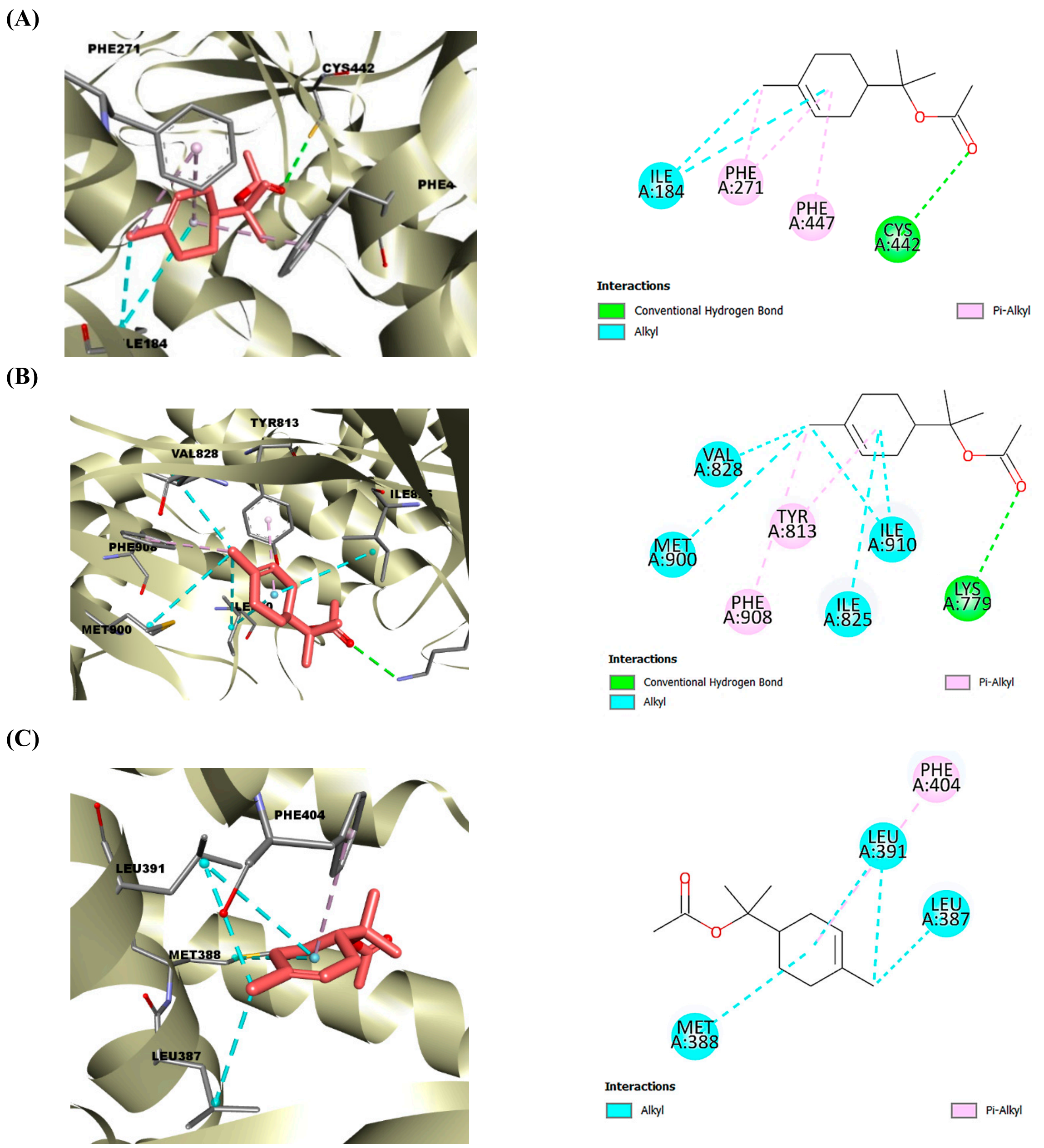

**(D)**

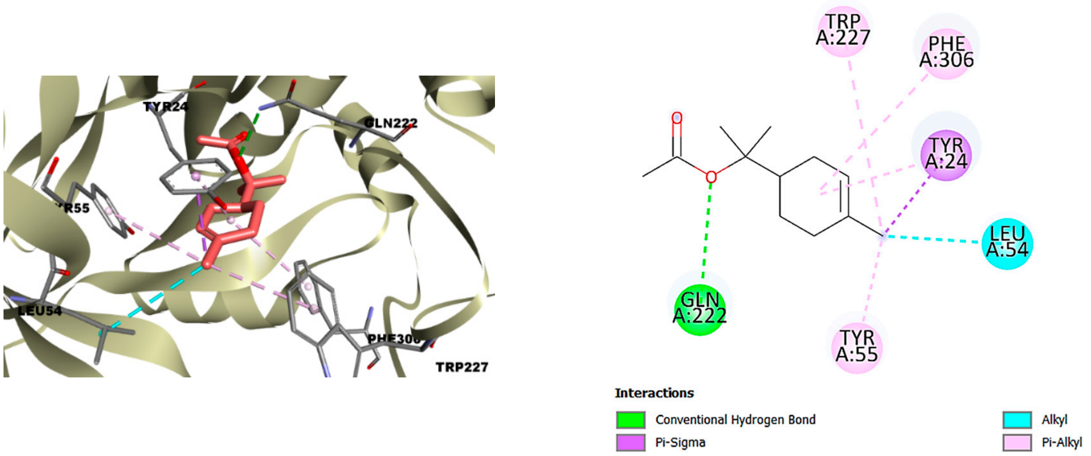


**(E)**

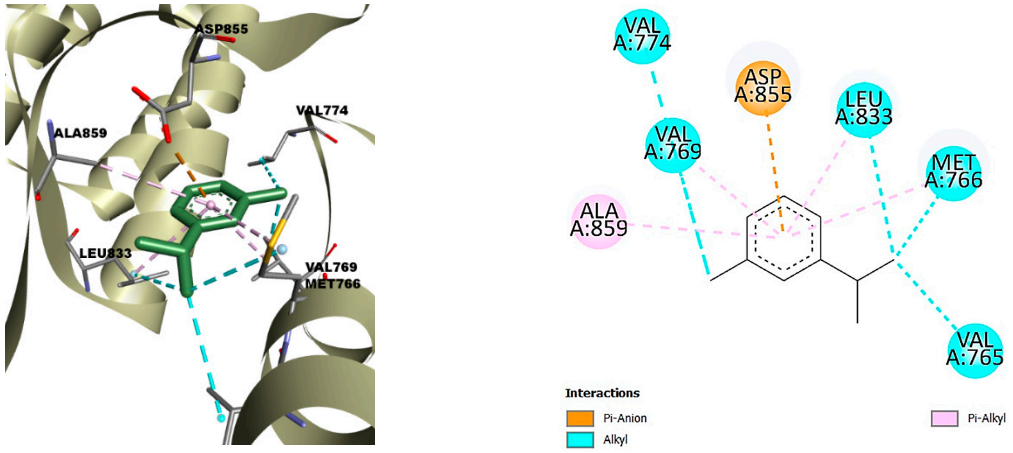


**(F)**

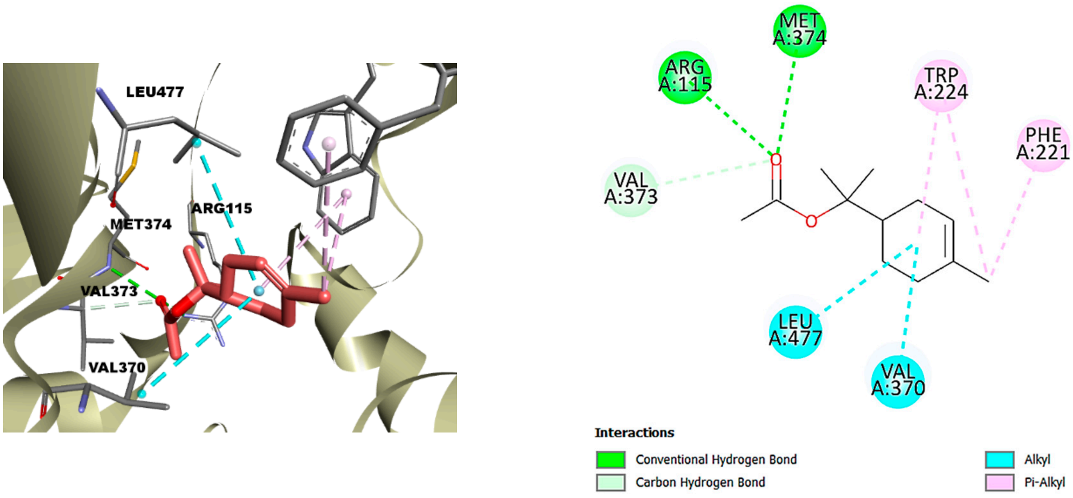


**Figure S4.** Illustrations of molecular docking results presented in both three-dimensional and two-dimensional formats. The interaction complexes shown include CYP3A4–*α*-Terpinyl acetate (A), PIK3CD–*α*-Terpinyl acetate (B), ESR1–*α*-Terpinyl acetate (C), AKR1C3–*α*-Terpinyl acetate (D), EGFR–*m*-Cymene (E), and CYP19A1–*α*-Terpinyl acetate (F).
