# Supplementary material for: Unveiling the Bioactive Efficacy of Cupressus sempervirens ‘Stricta’ Essential Oil: Composition, In Vitro Activities, and In Silico Analyses
Source: Pharmaceuticals (Basel). 2024 Aug 2;17(8):1019. doi: 10.3390/ph17081019 (PMC11357629; doi:10.3390/ph17081019)
Supplement: Supplementary file 1 [file pharmaceuticals-17-01019-s001.zip › Table S1-S2-S17.pdf]

Table S1. Inhibition effects of CSSLEO on α-Glucosidase enzyme activity.

CSSLEO conc. (µg/ml)	α-Glucosidase Enzyme Inhibition (%) (Mean ± SD)
500	71.83 ± 0.75
250	62.95 ± 0.13
125	54.74 ± 0.68
62.5	42.86 ± 1.02
31.25	35.09 ± 1.33
15.6	30.72 ± 0.54
7.8	23.18 ± 0.36
3.9	17.04 ± 0.62
2	11.82 ± 0.46
1	7.96 ± 0.32
0.5	4.87 ± 0.19
0.25	3.04 ± 0.38
0	0

Table S2. Inhibition effects of CSSLEO on α-amylase enzyme activity.

CSSLEO conc. (µg/ml)	α-Amylase Enzyme Inhibition (%) (Mean ± SD)
500	79.58 ± 0.64
250	72.17 ± 0.81
125	66.34 ± 0.62
62.5	62.89 ± 0.73
31.25	52.64 ± 0.98
15.6	44.13 ± 1.02
7.8	36.68 ± 0.74
3.9	30.45 ± 0.61
2	25.72 ± 0.46
1	19.39 ± 0.53
0.5	15.06 ± 0.42
0.25	12.35 ± 0.31
0	0

Table S17. Target proteins, the corresponding grid coordinates, and amino acid residues of the active sites.

Target protein	PDB ID	Grid box coordinates		Amino acid residues of the active site
		Centers (x, y, z)	Sizes (x, y, z)	
**CYP3A4**	1TQN	-18.752, -16.9648, -7.25041	31.2649, 40.8079, 31.3917	ILE50, TYR53, PHE57, ASP76, GLN78, GLN79, ASN104, ARG105, ARG106, PRO107, PHE108, GLY109, VAL111, ILE118, SER119, ILE120ALA121, GLU122, TRP126, ARG130, PHE137, SER180, VAL183, ILE184, THR185, THR187, SER188, VAL191, PHE203, THR207, ARG212, PHE213, PHE215, LEU216, PHE220, LEU221, ILE223, THR224, PRO227, ILE230, PHE241, LEU249, SER252, VAL253, MET256, PHE271, LEU272, MET275, VAL296, SER299, ILE300, ILE301, PHE302, ILE303, PHE304, ALA305, GLY306, GLU308, THR309, THR310, SER312, VAL313, LEU364, PRO368, ILE369, ALA370, MET371, ARG372, LEU373, GLU374, ARG375, PRO434, PHE435, GLY436, ARG440, ASN441, CYS442, ILE443, GLY444, PHE447, ALA448, ASN451, MET452, GLY481, LEU482, LEU483, GLN484
**PIK3R1**	4JPS	-35.2651, -1.77963, 46.3288	22.893, 23.5458, 23.6223	TRP335B, ILE338B, GLU342B, GLU345B, LYS346B, SER429B, LYS430, ASP434B, GLN435B, VAL437B, LYS575B, ASP578B, GLN579, MET582B, TRP583B, GLN586B,
**PIK3CD**	6PYR	38.2656, 10.6543, 37.7611	32.7158, 31.9168, 21.3798	LYS708A, THR750A, PHE751A, MET752A, ASP753A, SER754A, LYS755A, MET756A, TRP760A, ILE777A, LYS779A, ASP782A, ASP783A, LEU784A, ASP787A, LEU791A, TYR813A, CYS815A, ILE825A, GLU826A, VAL827A, VAL828A, SER831A, ASP832A, THR833A, ALA835A, ASN836A, ILE891A, GLY892A, ASP893A, ARG894A, HIS895A, ASP897A, ASN898A, MET900A, PHE908A, ILE910A, ASP911A, PHE912A, GLY913A, HIS914A
**ESR1**	1SJ0	31.5624, -0.585796, 21.5005	21.8081, 24.0051, 29.2518	MET343, LEU346, THR347, LEU349, ALA350, ASP351, GLU353, LEU354, GLU380, TRO383, LEU384, LEU387, MET388, LEU391, ARG394, PHE404, MET421, ILE424, PHE425, LEU428, GLY521, MET522, HIS524, LEU525, TYR526,

				CYS530, VAL533, VAL534, PRO535, LEU536, LEU539
**AKR1C3**	4H7C	-2.5686, 4.95585, 11.8163	20.1372, 22.8914, 29.2066	GLY22, THR 23, TYR24, PRO26, ASP50, LEU54, TYR55, LYS84, TRP86, SER87, HIS117, SER118, MET120, LEU122, SER166, ASN167, GLN190, TYR216, SER217, ALA218, LEU219, SER221, GLN222, ARG223, ASP224, ARG226, TRP227, VAL228, LEU268, ALA269, LYS270, PHE306, ASN307, SER308, SER310, PHE311, HIS314, TYR317, PRO318, TYR319
**EGFR**	5U8L	3.09465, -13.4152, -37.878	33.4942, 34.9322, 34.2797	LEU718, SER720, GLY721, ALA722, PHE723, GLY724, VAL726, LYS728, ALA743, ILE744, LYS745, LEU747, ARG748, GLU749, ALA750, THR751, SER752, PRO753, LYS754, ASN756, ILE759, GLU762, ALA763, VAL765, MET766, VAL769, VAL774, CYS775, ARG776, LEU777, ILE780, LEU782, THR783, SER784, THR785, VAL786, LEU788, MET790, GLN791, LEU792, MET793, PRO794, GLY796, LEU828, LEU833, HIS835, ARG836, ASP837, AEG841, ASN842, LEU844, ILE853, THR854, ASP855, PHE856, GLY857, ARG858, ALA859, LYS860, LEU861, TYR 869, ALA871, GLU872, GLY873, GLY874, L857, ARG858, ALA859, LYS860, LEU861, TYR 869, ALA871, GLU872, GLY873, GLY874, LYS875, VAL876, MET881, SER885, ARG889, TYR891
**CYP19A1**	3S79	82.6707, 50.3025, 48.4605	28.4623, 31.4429, 29.7529	MET107, ARG115, ILE132, ILE133, PHE134, TRP141, ARG145, PHE148, LEU152, MET160, ARG192, SER199, PHE203, VAL214, ILE217, GLN218, PHE221, ASP222, TRP224, GLN225, GLU302, MET303, ILE305, ALA306, ALA307, PRO308, ASP309, THR310, MET311, VAL313, SER314, PHE317, MET318, SER363, MET364, ARG365, GLN 367, PRO368, VAL369, VAL370, LEU372, VAL373, MET374, ARG375, ILE398, GLY399, HIS402, PHE427, PRO429, PHE430, GLY431,

			ARG435, GLY436, CYS437, ALA438, GLY439, ILE442, ALA443, MET446, MET447, ILE474, LEU477, SER478, HIS480, PRO481, ASP482, GLU483, THR484
